# Discharge of deeply rooted fluids from submarine mud volcanism in the Taiwan accretionary prism

**DOI:** 10.1038/s41598-019-57250-9

**Published:** 2020-01-15

**Authors:** Nai-Chen Chen, Tsanyao Frank Yang, Wei-Li Hong, Tsai-Luen Yu, In-Tian Lin, Pei-Ling Wang, Saulwood Lin, Chih-Chieh Su, Chuan-Chou Shen, Yunshuen Wang, Li-Hung Lin

**Affiliations:** 1 Department of Geoscience, National Taiwan University, Taipei, Taiwan; 2 Geological Survey of Norway, Trondheim, Norway; 3 Research Center for Future Earth, National Taiwan University, Taipei, Taiwan; 4 Exploration and Development Research Institute, CPC, Taiwan; 5 Institute of Oceanography, National Taiwan University, Taipei, Taiwan; 6 Central Geological Survey, MOEA, Taipei, Taiwan

**Keywords:** Ocean sciences, Geochemistry

## Abstract

Qualitative and quantitative assessments of fluid cycling are essential to address the role and transport of deeply sourced fluids in subduction systems. In this study, sediment cores distributed across a submarine mud volcano (SMV) offshore southwestern Taiwan were investigated to determine the characteristics of fluids generated through the convergence between the Eurasian and Phillippine Sea Plates. The low dissolved chloride concentration combined with the enrichment of ^18^O, and depletion of ^2^H of pore fluids suggest the discharge of deep freshwater formed by smectite dehydration at an equilibrium temperature of 100 to 150 °C. The upward fluid velocities, decreasing from 2.0 to 5.0 cm yr^−1^ at the center to a negligible value at margin sites, varied with the rate and efficiency of anaerobic methanotrophy, demonstrating the impact of fluid migration on biogeochemical processes and carbon cycling. By extrapolating the velocity pattern, the flux of fluids exported from 13 SMVs into seawater amounted up to 1.3–2.5 × 10^7^ kg yr^−1^, a quantity accounting for 1.1–28.6% of the smectite-bound water originally stored in the incoming sediments. Our results imply that SMVs could act as a conduit to channel the fluids produced from great depth/temperature into seafloor environments in a subduction system of the western Pacific Ocean.

## Introduction

Fluid cycling is of great importance to magma and seismicity generation in subduction systems. The release of fluid from the subducting plate into the overlying wedge decreases the liquidus temperature of lithospheric materials, thereby increasing the degrees of partial melting and facilitating melt extraction for arc magmatism^1^. Such fluid circulation in the deep crustal region also leads to increasing the pore pressure and reactivity of minerals, modifying the rheological property and weakening the rock strength^1–3^. The discharge of deeply generated fluids into the bottom ocean further influences the distribution of biological communities and seawater chemistry^4,5^. Therefore, a systematic assessment of fluid origins and its intrinsic characteristics in subduction systems would facilitate the implementation of a quantitative framework for fluid cycling and budget.

Sediment compaction and mineral dehydration are the most important mechanisms for fluid generation and circulation in an accretionary prism^6,7^. In essence, both mechanisms are driven by the increasing pressure and temperature associated with sediment burial and deformation. However, the geochemical signatures inherited from individual mechanisms are drastically different. Porewater excluded by sediment compaction through burial and deformation bears a composition essentially identical to seawater (e.g., ~560 mM chloride, and δ^18^O and δ^2^H values around 0‰). In contrast, lattice-bound water released by mineral dehydration is salt-free and carries δ^18^O and δ^2^H values deviating from its sourced fluid (e.g., seawater)^8–10^. Volatile elements (e.g., B, and Li) desorbed from clay minerals are also commonly accompanied with mineral dehydration^11,12^. Fluids generated from different mechanisms are, however, susceptible to the mixing during migration, thereby obscuring the pristine signatures that could be used to constrain the source depth, physico-chemical parameters of reactions, and fluid budget. Elucidating the observed characteristics remains challenging and often requires a sampling strategy targeting specific geological features or structures.

As one of the prominent seafloor features, submarine mud volcanoes (SMVs) are commonly distributed in accretionary prisms where rapid sedimentation and tectonic interaction lead to the gravitational instability of unconsolidated shale/mudstone, fluid overpressurization, and formation of dense fracture array^9,12–14^. Because the fracture network associated with the plate convergence could be extended to as deep as the lower crust, SMVs are able to efficiently export deeply sourced fluids, sediments, and reducing volatiles to the seafloor, generating morphological domes and depressions, mudflows, and even biological hotspots with colonies depending on leaked gaseous hydrocarbons^8–10^. While SMVs provide a window to witness the deep subsurface characteristics, upward migrating hydrocarbon gases produced by thermal maturation or methanogenesis also drive the sulfate-dependent anaerobic oxidation of methane (AOM) at shallow depths^9,10^. Such a biological removal mechanism has been considered to be effective in maintaining a low level of methane in seawater^15^. Overall, the exact characteristics and quantities of deep fluids discharged from SMVs, and their impacts on biogeochemical activities remain poorly constrained in the subduction system of the western Pacific Ocean.

**Figure 1:**
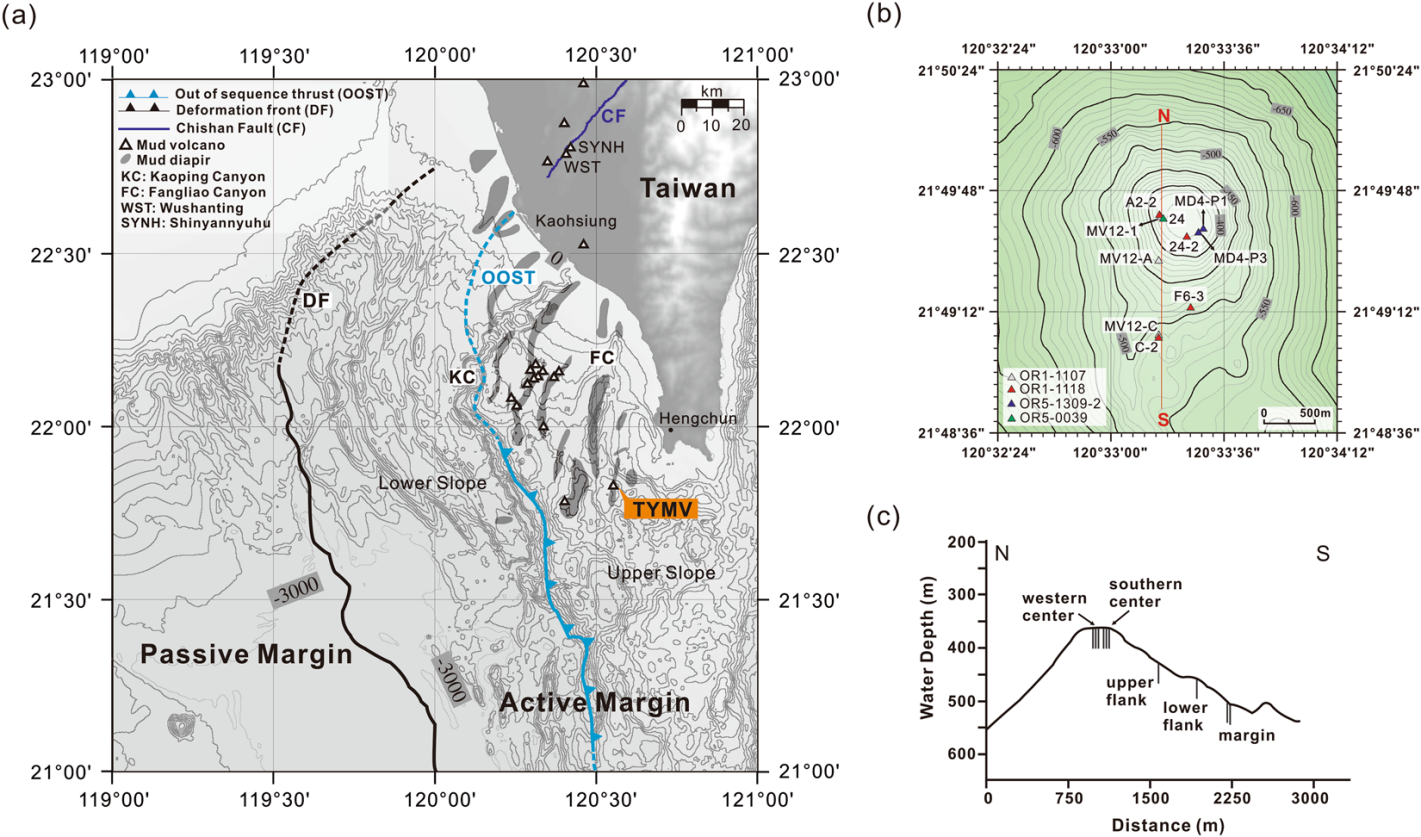
(**a**) Bathymetric map overlaid with the distribution of mud volcanoes^21,71^, mud diapirs^20^, geological structures^20,71^, and study site TY1 offshore southwestern Taiwan (the map was created by the open source GMT software^72^ using the NOAA public database^73^). (**b**) Enlargement of the map for coring sites on TY1. (**c**) A topographic profile for TY1 (N to S in b) with the core sites projected.

In this study, we present a comprehensive geochemical dataset of fluids extracted from sediment cores recovered from sites distributed across an SMV, TY1, in the accretionary prism offshore southwestern Taiwan (Fig. 1). These data were used to assess the fluid source, formation temperature, and fluid-rock ratio for the reaction in the source region. Reactive transport modeling was further applied to quantify the fluxes of fluid and methane exported from various sediment compartments to seawater. Finally, the possible fluid transport pathways in the Taiwan accretionary prism were discussed.

## Geological Settings

Offshore southwestern Taiwan is a westward extending accretionary prism related to the subduction of the Eurasian Plate underneath the Philippine Sea Plate since 18 Ma^16–18^. The deformation front and its southward extension, the Manila trench, defines the boundary between passive and active margins (Fig. 1a). The active margin is further divided into two structural domains separated by the out-of-sequence thrust: the Lower Slope and the Upper Slope domains^19^ (Fig. 1a). The Lower Slope domain is featured with the fold-and-thrust ridges; in contrast, the Upper Slope domain is characterized by mud diapiric structures with mud volcanoes ^20^. To date, 13 submarine mud volcanoes have been identified^21^. The largest one, Tsangyao Mud Volcano Group (TYMV; re-named after MV12^22^), is composed of two mud volcanoes (TY1 and TY2; Supplementary Fig. S1) fed by one mud diapir, as evidenced by seismic reflection profiles^21,23^. The TY1 has a conical structure and a wide flat top with a diameter of ~500 m at a water depth of ~370 m^21^ (Fig. 1b,c). Fluid/mud discharges occur at a frequency of every 3 to 10 seconds^24^. Two major gas plumes were detected on the crest by a multibeam echo sounder. These gas plumes could reach to a height of up to 367 m above the seafloor^24^. Other gas venting signals were also observed at the flank of the TY1 (Supplementary Information, Fig. S2).

## Results

### Fluid geochemistry

#### Variation of solute profiles across TY1

**Figure 2:**
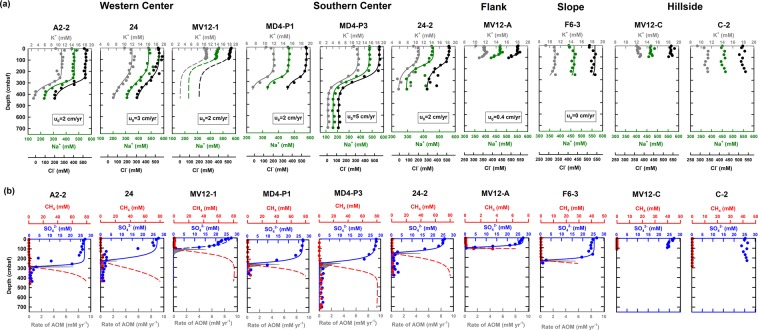
Concentration depth profiles and results of reactive transport modeling. (**a**) Chloride (in black), sodium (in green), and potassium (in gray) concentration depth profiles at ten sites investigated in this and previous studies (for sites MV12-A)^22^. The solid lines represent the best fit of the modeled results. The lower boundary conditions at site MV12-1 (in dashed line) were assumed to be the same as those at site A2-2. The modeled upward velocity was also provided. (**b**) Modeled results for sulfate (blue solid line) and methane (red dashed line) depth profiles and AOM rates (R_AOM_; gray area) at eight sites.

**Figure 3:**
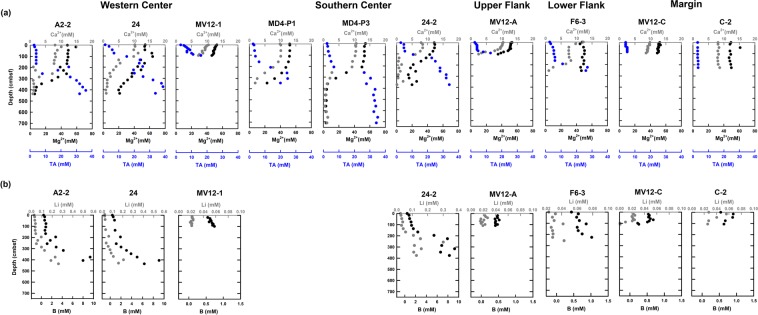
Cation abundance and total alkalinity profiles. (**a**) Calcium (in gray), magnesium (in black), and TA (in blue) concentration profiles at ten sites investigated in this and previous studies (for sites MV12-A)^22^. (**b**) Lithium (gray dot) and boron (black dot) concentration profiles at eight sites.

Profiles of solute and gas concentrations are shown in Figs. 2 and 3. Most porewater in the upper 120 to 280 cm of sediment had a seawater-like composition. Below this interval at the center sites, concentrations of most ions decreased with depth (potassium, sodium, magnesium, calcium, chloride, and sulfate). In contrast, concentrations of total alkalinity (TA), boron, and lithium increased with depth. Between 120 and 280 cm below seafloor (cmbsf), sulfate was undetectable and accompanied by an increase in methane concentration (1‒2 mM), forming a sulfate to methane transition zone (SMTZ). Changes in concentration gradient of other solutes were also observed across the SMTZ. For example, calcium and magnesium concentrations decreased to ca. 1 mM and lower than 5 mM below the SMTZ, respectively. The decrease was coincident with the increase in TA concentration (to around 35 mM).

At the upper flank (site MV12-A) and lower flank (site F6-3) sites, significant decreases in sulfate, calcium, and magnesium concentrations coincided with the increase in methane and TA concentrations (Figs. 2 and 3). Both lithium and boron concentrations remained at constant values throughout the core at the upper flank site. In contrast, boron and lithium concentrations increased with depth at the lower flank site, where SMTZ was observed at the bottom of the core.

At margin sites (sites MV12-C and C-2), concentrations of most ions were close to seawater values. In addition, methane concentrations only increased to at most 50 μM. Both boron and lithium concentrations at site MV12-C decreased with depth (Fig. 3b).

#### Porewater isotope data

**Figure 4:**
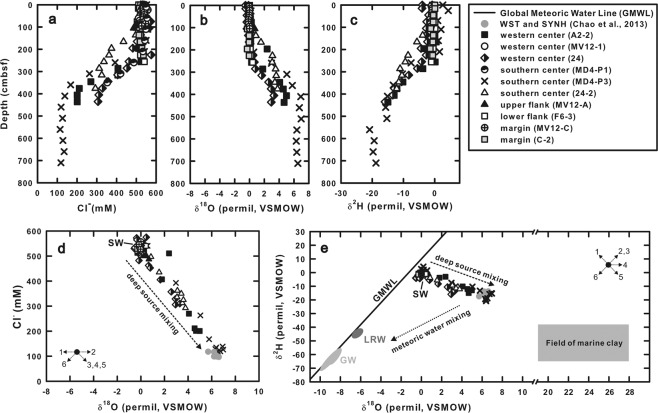
(**a–c**) Profiles of chloride concentrations and δ^18^O and δ^2^H values. (**d**,**e**): Plots of δ^18^O value versus chloride concentration and δ^18^O versus δ^2^H values. Gray dots are data from terrestrial mud volcanoes for comparison^74^. The black line in (**e**) indicates the Global Meteoric Water Line (GMWL; δ^2^H = 8 × δ^18^O + 10). Areas denote with “LRW” and “GW” in (**e**) marked the ranges of isotopic compositions for local meteoric water and groundwater from adjacent onshore areas, respectively^75^; gray square area represents the δ^18^O and δ^2^H values of marine clay^48–51^. Arrows marked with numbers in (**d**) and (**e**) represent processes potentially occurring at the source depth, and their directions denote the trends associated with the processes^8^: 1: volcanic ash alteration at temperatures lower than 300 ^o^C; 2: volcanic ash alteration at temperatures higher than 300 ^o^C; 3: gas hydrate dissociation; 4: biogenic opal recrystallization; 5: clay mineral dehydration; 6: meteoric water input.

Profiles of δ^18^O and δ^2^Η values of porewater are shown in Fig. 4. At center sites, δ^18^O and δ^2^Η values of porewater were close to 0‰ at either above 120 or 280 cmbsf, and increased and decreased, respectively, further down core. The data for the deepest sample deviated from the global meteoric water line (GMWL) in a way that δ^18^O values increased to +6 to +7‰ while δ^2^Η values decreased to −20 to −12‰ (Fig. 4d,e). At the upper flank (site MV12-A) and lower flank (site F6-3) sites, only slight variations in δ^18^O and δ^2^H values were observed at the bottom of the core (δ^18^O value increased to +0.7‰ and δ^2^H value decreased to −1.1‰). At the margin sites (sites MV12-C and C-2), δ^18^O values were close to 0‰ throughout the core, while δ^2^H values decreased slightly to −1.4‰ at the bottom of the core.

### Hydrocarbon gases and carbon isotopic compositions

**Figure 5:**
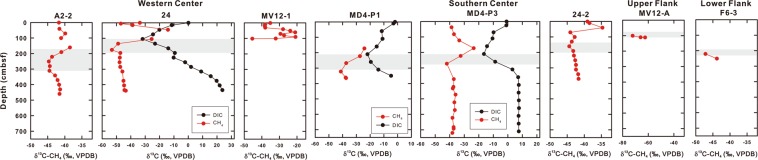
Profiles of δ^13^C-CH_4_ (in red) and δ^13^C-DIC values (in black). Gray shadows represent the SMTZ at each site.

At the western and southern center sites, δ^13^C-CH_4_ values increased from −40‰ on the seafloor to −20‰ above the SMTZ. The δ^13^C-CH_4_ value was the smallest at the SMTZ (−50 to −45‰) and remained at a constant value below the sulfate reduction zone (Fig. 5). At the upper flank (site MV12-A) and lower flank (site F6-3) sites, the δ^13^C-CH_4_ value was the smallest at the SMTZ (−70‰ at site MV12-A and −48‰ at site F6-3). The δ^13^C-DIC (dissolved inorganic carbon) patterns for center and flank sites were similar in a way that the values were the smallest at the SMTZ (−30‰ at site 24; −20‰ at sites MD4-P3 and MD4-P1) and increased towards the seafloor (~0‰) and the bottom of the cores.

**Figure 6:**
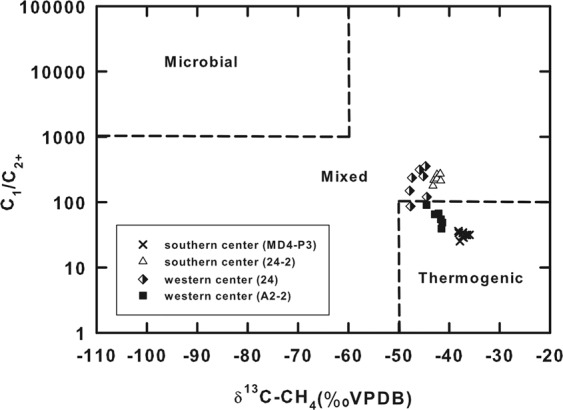
Plot of δ^13^C-CH_4_ value versus C_1_/C_2+_ ratio for samples collected at center sites. The assignment of gas origin is based on Bernard *et al*.^76^.

The C_1_/C_2+_ ratios and the δ^13^C-CH_4_ values for sites A2-2 and MD4-P3 were between 25 and 90, and between −35.9 and −44.5‰, respectively, falling in the region of thermogenic methane in the plot of C_1_/C_2+_ ratio versus δ^13^C-CH_4_ value (Fig. 6). For sites 24 and 24-2, the δ^13^C-CH_4_ values were between −48 and −37‰, whereas the C_1_/C_2+_ ratios increased to more than 120. These data points fell in a region between microbial and thermogenic methane.

### Reactive transport modeling and fluid flux from SMVs

The reactive transport modeling yielded that upward fluid velocities at western and southern center sites were in the range of 2 to 3 and 2 to 5 cm yr^−1^, respectively (Fig. 2). Upward fluid migration is likely absent at the margin site as the solute concentration do not change significantly with depth. The total fluid discharges from the craters and the rest of the cone structures of 13 SMVs were calculated to be 2.9 to 7.3 × 10^6^ kg yr^−1^ and 1.0 to 1.7 × 10^7^ kg yr^−1^, respectively (Supplementary Table S6).

The modeled methane fluxes beneath the depth of SMTZ at the western and southern centers were in the range of 1020‒1530 and 972‒2990 mmol m^−2^ yr^−1^, respectively (Table 1). The methane fluxes above the SMTZ at these center sites were 162‒1380 mmol m^−2^ yr^−1^. The methane flux beneath and above the depth of SMTZ was much lower (347‒456 and 9‒47 mmol m^−2^ yr^−1^, respectively) at the flank sites (sites MV12-A and F6-3) than at the center sites. While the methane fluxes above and beneath the SMTZ decreased with the distance from the center, the ratios (flux beneath SMTZ/flux above SMTZ) were relatively higher at the flank sites than at the center sites. Such a variation was translated into an increase in AOM efficiency from 54% at the center sites to 98% at the lower flank sites (Table 1).

**Table 1:** Microbial activity derived from reactive transport modeling.

	Unit	Western center			Southern center			Upper flank	Lower flank
		A2-2	24	MV12-1*	MD4-P1	MD4-P3	24-2	MV12-A	F6-3
Rate of anaerobic methanotrophy	mmol m^−2^ yr^−1^	858	861	872	943	1610	740	300	447
Rate of organic sulfate reduction	mmol m^−2^ yr^−1^	40.5	31.8	14.2	33.6	4.80	19.9	14.5	34.4
Rate of methaogenesis	mmol m^−2^ yr^−1^	24.2	32.9	52.2	20.0	68.0	36.6	1.78	5.57
Methane flux from depth	mmol m^−2^ yr^−1^	1020	1530	1150	1280	2990	972	347	456
AOM-filteration efficiency	%	84	56	76	74	54	76	86	98

*Parameters (core length and lower boundary conditions) are assumed to be the same as those for site A2-2 (Supplementary Table S4).

## Discussion

The decreasing chloride concentration with depth (Fig. 4a) and carbon isotopic composition and abundance patterns of methane (Figs. 5 and 6) suggest an input of freshwater and thermogenic methane from great depth. Crossplots among δ^18^O values, δ^2^H values, and chloride concentrations (Fig. 4e) indicate the deviation of the fluid compositions for the center sites from the GMWL and seawater. The geochemical variation could be best accounted for by the impact of seawater and a deep fluid characterized by low chlorinity, high δ^18^O values, and low δ^2^H values. Such variations in δ^18^O and δ^2^H values cannot be explained by gas hydrate dissociation, ash alteration at high or low temperature, or biogenic opal recrystallization, as these reactions enrich fluids with ^2^H or cause no fractionation on hydrogen isotopes^8,10^. Clay dehydration appears to be the most plausible mechanism to account for the observed geochemical signature.

To approximate the temperature of fluid formation, specific cation-ratios were used to derive the fluid temperature^25–27^. The validity of geothermometers lies in the requirement of the presence of reactant mineral. Since sediments or mineralogy in the source region are not available, the constituent mineral for the formation currently outcropping in southwestern Taiwan was first adopted to evaluate the applicability of the specific geothermometer. The inference is based on the fact that the subduction of the Eurasian Plate underneath the Philippine Sea Plate generated a series of westward propagating thrusts that sequentially exhumed the sediments once deposited offshore Taiwan^18^. Therefore, the strata on land could mirror the characteristics and constituents of sediments in the potential source region that is accessible only with the deep drilling. The Plio-Pleistocene *Gutingkeng Formation* formed in shelf break environments was chosen as the analogy for the source material. The formation is primarily composed of mudstone with lens of siltstone and sandstone^28^. Feldspars at abundances of up to 5% in this formation have been observed^29^. In addition to the constraint from the formation in terrestrial environments, feldspars in cores retrieved from the ODP 1144 site in the abyssal region of the South China Sea amounted up to 1.33 wt.% of the total sediments^30^. Therefore, both observations support the possibility that feldspars are available in the source region and serve the source of sodium and potassium in fluids. The geothermometer based on the Na/Li and Na/K ratio were used for the temperature estimation^25,27,31^:

1 $$T(^\circ {\rm{C}})=\frac{1195}{log(\frac{Na}{Li})-0.13}-273$$

2 $$T(^\circ {\rm{C}})=\frac{1170}{log(\frac{Na}{K})+1.42}-273$$

where the concentration is in molality in Eq. (1) and mg/kg in Eq. (2). The Ca-Mg-K and Mg/Li geothermometers were not considered because the porewater chemistry for all depths at the TY1 is oversaturated with respect to a number of carbonate minerals, such as dolomite and Mg-calcite (Supplementary Table S1). Furthermore, the concentration of magnesium is lower than what is expected by the dilution of seawater by freshwater alone, again suggesting the potential removal of magnesium by carbonate precipitation (Supplementary Fig. S3). The SiO_2_ geothermometers were not applied due to the potential underestimate caused by rapid cooling through the intrusion of the overlying seawater^26^.

The fluid temperature was calculated to range between 100 and 150 °C. Our estimated temperature is supported by the high concentrations of lithium and boron (Fig. 3b), a pattern consistent with the results from laboratory experiments by which lithium and boron are preferentially released from silicate minerals in marine sediments starting at ca. 50 °C^32–34^. The estimated temperature is also consistent with the predominance of thermogenic methane over microbial methane as being inferred on the basis of isotopic compositions and abundance ratios (Fig. 6). Furthermore, the geothermal gradients measured at TY1 vary from 25 °C/km at the margin sites to 390 °C/km at the center sites^23^. Since the crater is directly connected with the conduit channeling hot fluids from great depth, the measured geothermal gradient would be distorted toward a higher value by the advective fluid flow^23,35^. Instead, the averaged value^35^ (25 °C/km) measured at the margin sites and in surface sediments in the region was chosen to represent the regional geothermal gradient. Using this value, the depth producing the observed geochemical signatures was calculated to range between 3.6 and 5.7 kmbsf (kilometer below seafloor).

These lines of evidence indicate that a significant fraction of pore fluid is contributed from clay dehydration at high temperatures. Illite and chlorite, which are considerably abundant in sediments offshore southwestern Taiwan^36^, are excluded because illite and chlorite are dehydrated at a much higher temperature range (>237 °C for illite^37^ and >650 °C for chlorite^38^). Kaolinite is also excluded as a result of being less abundant in the northern South China Sea^39^ and stable over the temperature range estimated in this study (decomposed at >800 °C and 19 GPa^40^). Since the transformation of smectite to illite proceeds at a temperature higher than 60 °C^8,34^ and produces fluids enriched in ^18^O and depleted in ^2^H, smectite is considered to be the most plausible mineral candidate to account for the observed geochemical signature. As abundant smectite and mixed layer (1.1‒24.8 wt.% in bulk sediments) have been found in the active margin^41^ and passive margin off southwestern Taiwan^39,42^, and sediments off the Luzon Arc^43–46^ (ODP, sites 1144 and 1146), smectite sourced from Taiwan orogeny and Luzon Arc volcanism and later being subducted/accreted as part of the Taiwan accretionary prism is the most likely source.

**Figure 7:**
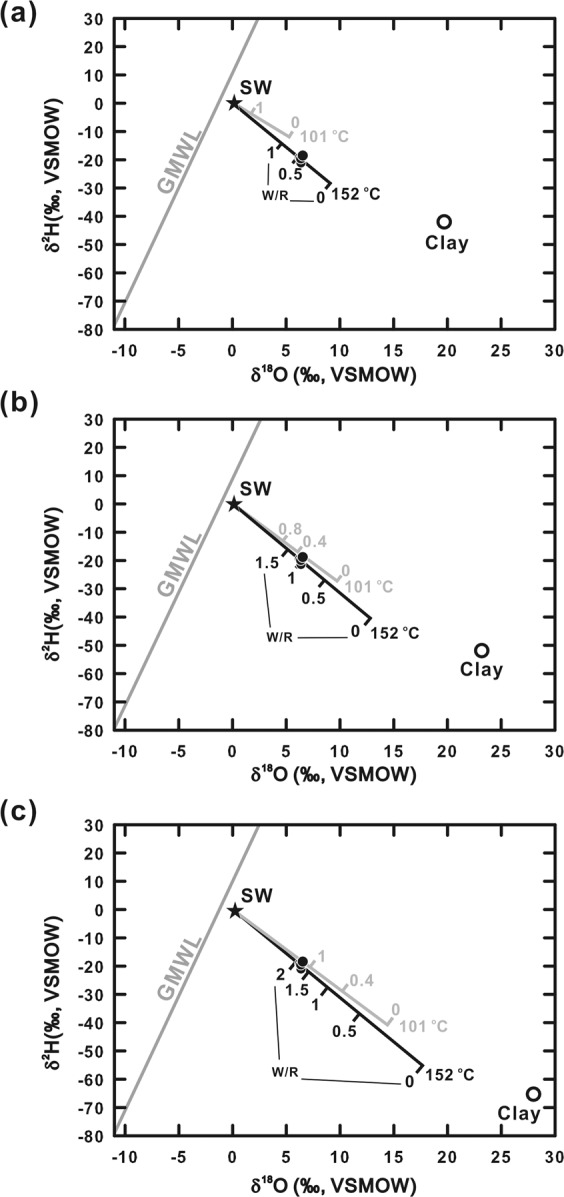
Isotopic compositions of fluids under different fluid-rock ratios (W/R) at different temperatures. The star symbol represents the seawater value (δ^18^O = 0‰; δ^2^H = 0‰). The gray line is the global meteoric water line (GMWL). Data obtained from the bottom part of site MD4-P3 are plotted as black dots. The open circles denote δ^18^O^i^ and δ^2^H^i^ values of smectite used in each case: (**a**) δ^18^O^i^-rock = +19‰; δ^2^H^i^ of rock = −39‰. (**b**) δ^18^O^i^-rock = +23‰; δ^2^H^i^ of rock = −51‰. (**c**) δ^18^O^i^-rock = +28‰; δ^2^H^i^ of rock = −66‰.

To further assess the smectite dehydration, we adopted an isotopic mass balance to calculate the ratio of water to rock (W/R) for reactions considering the exchange of oxygen and hydrogen between minerals and pore fluid under a closed-system condition^47^. The fluid-to-mineral ratio for the reaction was calculated based on the mass balance for hydrogen and oxygen isotope compositions^47^:

3 $${W}_{o}{\delta }^{18}{O}_{pw}^{i}+{R}_{o}{\delta }^{18}{O}_{r}^{i}={W}_{o}{\delta }^{18}{O}_{pw}^{f}+{R}_{o}{\delta }^{18}{O}_{r}^{f}$$

4 $${W}_{H}{\delta }^{2}{H}_{pw}^{i}+{R}_{H}{\delta }^{2}{H}_{r}^{i}={W}_{H}{\delta }^{2}{H}_{pw}^{f}+{R}_{H}{\delta }^{2}{H}_{r}^{f}$$

where *W*_*O*_ and *W*_*H*_ are moles of oxygen and hydrogen, respectively, in porewater; and *R*_*O*_ and *R*_*H*_ are moles of oxygen and hydrogen, respectively, in target minerals. The superscripts *i* and *f* represent the initial and final isotopic compositions, respectively. The subscripts *r* and *pw* denote isotopic compositions of rock and porewater, respectively. By rearranging Eqs. (3) and (4), ${\delta }^{18}{O}_{pw}^{f}$ and ${\delta }^{2}{H}_{pw}^{f}$ values were calculated using Eqs. (5) and (6):

5 $${\delta }^{18}{O}_{pw}^{f}=\frac{\frac{{W}_{O}}{{R}_{O}}{\delta }^{18}{O}_{pw}^{i}-{\Delta }_{r-w}^{O}+{\delta }^{18}{O}_{r}^{i}}{1+\frac{{W}_{O}}{{R}_{O}}}$$

6 $${\delta }^{2}{H}_{pw}^{f}=\frac{\frac{{W}_{H}}{{R}_{H}}{\delta }^{2}{H}_{pw}^{i}-{\Delta }_{r-w}^{H}+{\delta }^{2}{H}_{r}^{i}}{1+\frac{{W}_{H}}{{R}_{H}}}$$

where ${\Delta }_{r-w}^{O}$ and ${\Delta }_{r-w}^{H}$ are isotopic fractionations of oxygen and hydrogen isotopes, respectively, during the exchange of isotopes between rock and porewater (Δ = 10^3^ ln α; α is the fractionation factor). The W/R ratios for oxygen and hydrogen are assumed to be identical (W_O_/R_O_ = W_H_/R_H_). A value of 0‰ was assigned for both the ${\delta }^{2}{H}_{pw}^{i}$ and δ^18^O^i^_pw_ values based on the bottom water measurements. The ${\delta }^{18}{O}_{r}^{i}$ and ${\delta }^{2}{H}_{r}^{i}$ values for marine clays range between +15.0 and +28.5‰ and between −80 and −4.1‰, respectively^48–51^. The ${\delta }^{18}{O}_{r}^{i}$ and ${\delta }^{2}{H}_{r}^{i}$ values were assumed to range from +19 to +28‰ and from −80 to −36‰, respectively. The temperature dependence for the fractionation factors for oxygen and hydrogen isotopes was calculated according to previous studies for a temperature range between 100 and 150 °C^52,53^. The calculations yielded the δ^18^O and δ^2^H values varying as a function of equilibrium temperature and W/R ratio (Fig. 7). Using the measured isotopic compositions of the porewater collected from the center sites (site MD4-P3, Fig. 1b), the W/R ratios were estimated to be <0.5, 0.3–0.9, and 1.1–1.9 if the ${\delta }^{18}{O}_{r}^{i}$ values were set as +19‰, +20 to +22‰, and +23 to +28‰, respectively.

The amount of needed smectite could be further deduced from these W/R ratios considering the possible volume of porewater and assuming the complete reactivity of all available porewater and smectite. To constrain the possible porosity at the depth estimated for smectite dehydration, the porosity variation for cores collected from the Nankai Trough^54^ (IODP 358, site C0002, 3.2-km long) was used for the extrapolation. The measurement from IODP 358 yielded a porosity of 17% at the depth of 3.2 kmbsf ^54^. By applying the exponential reduction function, the porosity at 5.7 kmbsf was calculated to be 5.6% (Supplementary Table S2). Assuming that the porespace is saturated with porewater and that the porosity is 6%, the quantity of porewater would be lower than 3 wt.% of the bulk sediment. To fulfill the estimated W/R ratios (0.3–1.9) for smectite dehydration, 1.6–10 wt.% of smectite is needed. Such a mineral abundance is consistent with the measured amount of smectite in the current active and passive margin^41,43–45^ (1.1 to 6.2 wt.% in sediments collected by a sediment trap deployed southwestern Taiwan; 4.5 to 24.8 wt.% in sediments from the ODP 1144 and 1146).

As subducted-smectite could be the source of freshwater, fluid fluxes in the SMV region of the accretionary wedge were further assessed. The total amount of smectite-bound fluid within the incoming plate was estimated considering the weight of the incoming plate and the content of smectite and porosity in the sediments^55–57^:

7 $${F}_{a}={C}_{a}\cdot H\cdot (1-{\varphi }_{a})\cdot \rho \cdot {V}_{a}\cdot {L}_{a}$$

where *F*_*a*_ is the flux, *C*_*a*_ is the weight percentage of mineral-bound water in smectite, *H* is the pre-subducted sediment thickness, *ρ* is the density of dry sediments, *φ*_*a*_ is the porosity of sediments from the incoming plate, *L*_*a*_ is the length of trench, and *V*_*a*_ is the subduction rate. The details for calculations were stated in Supplementary Information. The total smectite-bound fluid from the incoming plate was calculated to be 0.7–8.8 × 10^8^ kg yr^−1^.

As sediments are subducted, the buried seawater-like pore fluid and produced smectite-bound fluid would be squeezed and lost gradually with the increasing temperature and pressure. As a result, the actual amount of water released into SMVs region would be substantially lower than that from the incoming plate. Of the overall flux of brackish fluid expelled from SMVs offshore Taiwan (1.3–2.5 × 10^7^ kg yr^−1^), approximately 80% of this flux could be attributed to the smectite-bound fluid (1–2 × 10^7^ kg yr^−1^) considering the mixing of seawater and freshwater end components (based on the chloride concentration, 110 mM, at the bottom of site MD4-P3). This quantity of smectite-bound fluid released from the SMVs in the region constituted about 1.1–28.6% of the smectite-bound fluid originally subducted into the trench (0.7–8.8 × 10^8^ kg yr^−1^).

Previous modeling study using the data integrated from geophysical and mineralogical observations for the Nankai Trough^6^ demonstrated that the majority of the smectite-bound fluids (4.17 × 10^8^ kg yr^−1^; 88% of total smectite-bound fluids in the incoming plate) would be lost to the frontal prism, leaving a total of 0.56 × 10^8^ kg yr^−1^ smectite-bound fluids further released into the overlying wedge as the descending sediments reach the region beneath the Kumano basin where mud volcanoes are abundant. If such a fluid budget for the Nankai system is directly applied to the Taiwan subduction system, a total of 0.8–10.4 × 10^7^ kg yr^−1^ ( = 0.7–8.8 × 10^8^/ (4.17 × 10^8^/ 88%/ 0.56 × 10^8^)) smectite-bound fluids would be released from the descending sediments into the wedge in the Upper Slope. This estimated flux is comparable with the flux of freshwater expelled into seawater through SMVs offshore Taiwan obtained in this study (1–2 × 10^7^ kg yr^−1^). Whether such an analogy of the fluid budget could be drawn directly remains to be explored even though the plate geometry, heat flux, mineral type and content, convergence rate, and the distance between trench to mud volcanoes are comparable between the Nankai and Taiwan subduction systems (Supplementary Table S3).

The impact of the deep fluid on shallow subsurface microbial processes is reflected in geochemical profiles. The decreasing rate of upward fluid flow from the center towards the margin sites (Fig. 2) varied concomitantly in a similar and an opposite trend with the modeled AOM rates and efficiency, respectively (Table 1 and Supplementary Table S5). For example, at site MD4-P3 where the upward velocity was the greatest, the AOM rate was the highest among eight sites (1610 mmol m^−2^ yr^−1^), and the AOM efficiency was the lowest (54%) (Table 1). In contrast, the upward fluid flow was absent at the lower flank site, whereas the AOM efficiency reached 98% (Table 1). Such an observation suggests that the upward velocity of the fluid is the primary control of AOM rates. As the capacity of AOM is limited, the efficiency of methane removal could not accommodate the extremely high supply of methane at center sites. This pattern is consistent with those obtained in onshore mud volcanoes in Taiwan^22,58^ and SMVs elsewhere (e.g., the Hakon Mosby SMV^59^).

## Conclusions

Porewater and gases for cores distributed along a transect of TY1 were systematically analyzed to assess the spatial variation of fluid velocities and biogeochemical activities and further constrain the fluid budget. The co-variation of the isotopic composition and chloride concentration demonstrates that the majority of fluid retrieved from the crater is derived from smectite dehydration at a temperature of 100 to 150 °C or a depth of 3.6 to 5.7 kmbsf. Assuming that the isotopic equilibrium is reached, the water-rock interaction proceeds at a water-rock ratio of more than 0.3. Reactive transport modeling for compounds inert to microbial processes yielded that the velocity of upward migrating fluid varied from 2 to 5 cm yr^−1^ at the center sites to a negligible level at the margin sites. By extrapolating such a variation pattern for other SMVs offshore Taiwan, the overall flux discharged from SMVs ranged from 1.3 to 2.5 × 10^7^ kg yr^−1^. This fluid quantity accounts for 1.1–28.6% of the smectite-bound water originally stored in the incoming sediments, implying that SMVs could act as a conduit to channel the fluid produced from great depth/temperature into seafloor environments. The flux variations across the transect of a mud volcano strongly impact the magnitude and efficiency of the biological filtration of methane through methanotrophy.

## Methods and Materials

### Sampling sites

Four expeditions were conducted in the offshore southwestern Taiwan by R/V Ocean Researcher 5 (legs 1309–2 and 0039) and R/V Ocean Researcher I (legs 1107 and 1118). Piston and gravity cores with lengths ranging from 102 to 720 cm were recovered along a transect from the center to the margin of the TY1 cone structure (Fig. 1c). Sampling sites are listed in Table 2 and shown in Fig. 1b,c.

**Table 2:** Information of coring sites.

Cruise	Site	Longitude	Latitude	Location
OR5-1309-2	MD4-P3	120^o^ 33.46′	21^o^ 49.59′	southern center
OR5-1309-2	MD4-P1	120^o^ 33.49′	21^o^ 49.62′	southern center
OR5-0039	24	120^o^ 33.28′	21^o^ 49.66′	western center
OR1-1107	MV12-1	120^o^ 33.28′	21^o^ 49.67′	western center
OR1-1107	MV12-A	120^o^ 33.27′	21^o^ 49.47′	upper flank
OR1-1107	MV12-C	120^o^ 33.26′	21^o^ 49.08′	margin
OR1-1118	A2-2	120^o^ 33.48′	21^o^ 49.63′	western center
OR1-1118	24-2	120^o^ 33.28′	21^o^ 49.66′	southern center
OR1-1118	F6-3	120^o^ 33.34′	21^o^ 49.24′	lower flank
OR1-1118	C-2	120^o^ 33.27′	21^o^ 49.07′	margin

### Sampling and analytical methods

Sediments and core top seawater for gas analyses were collected and transferred into serum bottles. These bottles were later filled with saturated NaCl^60^ (in cruises OR5-1309-2 and OR5-0039) or 1 N NaOH^58^ (in cruises OR1-1107 and OR1-1118) as preservatives, sealed with butyl rubber stoppers, crimped with aluminum caps, and stored upside down at room temperature or 4 °C. Pore fluid samples for aqueous geochemistry were obtained through centrifugation and subsequently filtered through 0.22-µm nylon membrane syringe filters. The filtrates were further split into five fractions for the analyses of anion, cation, total alkalinity (TA), carbon isotopic compositions of DIC, and oxygen and hydrogen isotopic composition, and stored at 4 °C. For the cation samples, concentrated nitric acid (70%) was added at a volume ratio of 1:45 to preserve the valence state for elements sensitive to the redox change.

The concentrations of headspace hydrocarbon gases were determined by a gas chromatography (GC; SRI 8610 C and Agilent 6890 N) with the precision typically better than 5%^22,58^. Ion concentrations were determined by an ion chromatography (IC, 882 Compact IC Plus) equipped with a Metrosep A Supp 5 column for anions and a Metrosep C 4 column for cations and with the detection limit around 0.1 ppm in weight^61^. Lithium and boron were analyzed by a sector field inductively coupled plasma mass spectrometry (SF–ICP–MS) for ^7^Li and ^11^B at low resolution (M:ΔM ~300). Six standards were prepared from reference material NASS–5 (all from National Research Council Canada) with the uncertainty better than 3%. TA was determined by titrating the sample with 0.02 N HCl while bubbling with nitrogen^62^. Carbon isotopic compositions of methane and DIC were measured by an isotope ratio mass spectrometry (IRMS) in line with a gas chromatography and a combustion oven. Hydrogen and oxygen isotope compositions of porewater were measured by a LGR Liquid-Water Isotope Analyzer (LWIA; for cruises OR5-1309-2 and OR5-0039) and a Picarro L2140-i Analyzer (for cruises OR1-1107 and OR1-1118). The obtained isotopic compositions were expressed as the δ notation referenced to standards [δ = (R_sample_/R_std_ − 1) × 1000‰, where R is the ratio of heavy over light isotopes]. Standards are the Vienna Pee Dee Belemnite (VPDB) for carbon isotope, and the Vienna Standard Modern Ocean Water (VSMOW) for hydrogen and oxygen isotopes. The precisions are ±0.3‰ for δ^13^C values by the IRMS measurements, ±0.2‰ for δ^18^O values and ±2.0‰ for δ^2^Η values by the LWIA measurements, and ±0.03‰ for δ^18^O values and ±0.22‰ for δ^2^Η values by the Picarro spectroscopic measurements. The water content of cored sediments was calculated based on the weight loss of the sediments after freeze-drying. The porosity was converted from the water content assuming a dry sediment density of 2.7 g cm^−3^ ^63^.

### Reactive transport modeling

To deduce the velocity of upward fluid in such a dynamic environment^64^, a simplified one-dimensional reactive transport modeling was applied for porewater geochemistry profiles with a revised code^65^. The concentration-depth profiles of five dissolved species, including chloride, sodium, potassium, sulfate, and methane, were calculated. This model is formulated by a partial differential equation for solute transport and reactions^65,66^. The modeling was first performed on conservative solutes (chloride and sodium) exempted from microbial processes and most abiotic reactions in shallow sediment environments to determine the range of upward advection rates. The sodium and chloride concentrations in the shallow sediments (the top 120 to 280 cm of sediments; Fig. 2) did not vary much and cannot be attributed to bioirrigation^67^. Therefore, irrigation induced by bubble transport was incorporated to explain such constant concentrations^60,68^. Microbial reactions involving sulfate and methane were considered^12,65^. A detailed description of model-construction, chosen parameters, and boundary condition in the model along with additional sensitivity tests are given in the Supplementary Information.

### Fluid discharge from SMVs

To estimate the total discharge of brackish water from SMVs, the upward fluid velocities across the TY1 were compiled first with those obtained in the previous study^22^. The upward fluid velocities generally decreased with increasing distance from the edge of crater. Such a trend is also found in other SMVs in offshore Norway and the Gulf of Cadiz^12,69^ (such as the Håkon Mosby and Carlos Ribeiro SMVs). To generalize the fluid velocities across the entire cone structure, the variations in velocity with distance from the edge of the crater were fitted with the exponential equation (Supplementary Information, Fig. S9a). The calculation was based on the assumption that the fluid flow was confined through porespace and driven by a single, pressurized fluid source.

The velocity distribution was further integrated with the ring area coverage (Supplementary Information, Fig. S9b) to derive the total brackish water discharge using the following equation^12^:

8 $$\begin{array}{c}{\rm{Brackish}}\,{\rm{water}}\,{\rm{discharge}}\,({\rm{kg}}\,{{\rm{yr}}}^{-1})\\ \,=\,{\rm{porosity}}\times {\rm{area}}({{\rm{m}}}^{2})\times {\rm{upward}}\,{\rm{fluid}}\,{\rm{velocity}}\,({\rm{m}}\,{{\rm{yr}}}^{-1})\times {\rm{density}}({\rm{kg}}\,{{\rm{m}}}^{-3})\end{array}$$

In this calculation, the crater radius of 250 m, the cone diameter of 3600 m^21^, and the porosity of 36% (an average value of the porosity of TY1; Supplementary Fig. S6) were used. The density of water was assumed to be 1000 kg m^−3^. The same approach was applied to other 12 SMVs assuming that the upward velocity at the crater of individual SMVs was the same as that at TY1. Since each SMV has different diameters of its crater and cone structure, the relationship between the velocity and the distance ratio was constructed (normalized to the distance from the edge of crater). Such a relationship was then applied to calculate the discharge fluxes of other individual SMVs (Supplementary Information, Table S6). Terrestrial mud volcanoes were not considered because their sizes are too small when compared to SMVs^70^. More details are described in Supplementary Information.

## Supplementary information

supplementary information.

supplementary dataset.
