# Supplementary material for: Discharge of deeply rooted fluids from submarine mud volcanism in the Taiwan accretionary prism
Source: Sci Rep. 2020 Jan 15;10:381. doi: 10.1038/s41598-019-57250-9 (PMC6962449; doi:10.1038/s41598-019-57250-9)
Supplement: Supplementary file 1 — supplementary information. [file 41598_2019_57250_MOESM1_ESM.docx]

**Supplementary Information**

Discharge of deeply rooted fluids from submarine mud volcanism in the Taiwan accretionary prism

Nai-Chen Chen^1^, Tsanyao Frank Yang^1^, Wei-Li Hong^2^, Tsai-Luan Yu^1,3^, In-Tian Lin^4^, Pei-Ling Wang^5^, Saulwood Lin^5^, Chih-Chieh Su^5^, Chuan-Chou Shen^1,3^, Yunshuen Wang^6^, and Li-Hung Lin^1,3^

^1^Department of Geoscience, National Taiwan University, Taipei, Taiwan, ^2^Geological Survey of Norway, Trondheim, Norway, ^3^NTU Research Center for Future Earth, National Taiwan University, Taipei, Taiwan, ^4^ Exploration and Development Research Institute, CPC, Taiwan, ^5^Institute of Oceanography, National Taiwan University, Taipei, Taiwan, ^6^Central Geological Survey, MOEA, Taipei, Taiwan

**Contents of this file**

Figures S1 to S9

Tables S1 to S6

Thermodynamic calculations for the saturation state of authigenic carbonate

Ionic strength (I) was first calculated based on the concentrations of major and some minor ions. The calculation yielded I decreasing from around 0.6 to 0.7 for the top sediments (0 to 280 cmbsf) to 0.28 at core bottom. The Debye-Hückel Extended Law was further used to calculate activity coefficients ($\gamma_{i}$):

$log\gamma_{i}=\frac{-Az_{i}^{2}\sqrt{I}}{1+Ba_{i}\sqrt{I}}$ (S1)

where A and B are constants based on in situ temperature, $z_{i}$ is ion charge, $a_{i}$ is effective ionic radius, and $i$ represents each ion. The values of A, B, and $a_{i}$ were cited from Manov et al. (1943)^1^. Temperatures in sediments were calculated based on in situ bottom water temperature (10.5 ^o^C) and a temperature gradient of 0.39 ^o^C/m at TY1^2^. Density of porewater was assumed to be 1.03 g/cm^3^.

The activity quotients (Q) for the carbonate precipitation (reactions below) at site A2-2 were calculated using pH 8 and shown in Table S1. The solubility product constant (K_sp_) values were calculated using Eq. (S2):

Dolomite: Mg^2+^ + Ca^2+^ + 2HCO_3_^‒^ 🡪 CaMg(CO_3_)_2_ + 2H^+^ ΔG^o^ = 20.24

Mg-calcite: Mg^2+^ + HCO_3_^‒^ 🡪 MgCO_3_ + H^+^ ΔG^o^ = 29.5

Calcite: Ca^2+^ + HCO_3_^‒^ 🡪 CaCO_3_ + H^+^ ΔG^o^ = 11.54

$logK_{sp}=\frac{-\Delta G^{\circ}}{2.303RT}$ (S2)

where R is the ideal gas constant, and T is the in situ temperature.

Estimation of initial water budgets

The total amount of smectite-bound fluid within the incoming plate is estimated considering the weight of the incoming plate and the content of smectite and porosity in the sediments^7‒9^:

$F_{a}=C_{a}\cdot H\cdot(1-\varphi_{a})\cdot\rho\cdot V_{a}\cdot L_{a}$ (S3)

where $F_{a}$ is the flux, $C_{a}$ is the weight percentage of mineral-bound water in smectite, $H$ is the pre-subducted sediment thickness (1 km; 30% and 70% of sediments assumed to be accreted and subducted along with the plate, respectively^9^), $\rho$ is the density of dry sediments^10^ (2.7 g cm^-3^), $\varphi_{a}$ is the porosity of the sediments from the incoming plate^11^ (50%), $L_{a}$ is the length of trench (172 km; 43% of the whole trench length of 400 km covering the region with SMVs^12,13^), and $V_{a}$ is the subduction rate^3,6^ (66–76 mm yr^-1^). For seawater-like pore fluid, $\varphi_{a}$ instead of $(1-\varphi_{a})$ in Eq. (S3) was used. For smectite-bound fluid, smectite abundances at 4.5–24.8 wt.% for the passive margin^14‒17^ and abundances of mineral-bound fluid in smectite at 10 to 20% were adopted for calculation^18^. With these parameters, $C_{a}$ was calculated to be 0.5 – 5.0 wt.%. For seawater-like pore fluid, the parameters include $\rho$ (1.03 g/cm^3^) and $C_{a}$ (100 wt.%). The total smectite-bound fluid and seawater-like pore fluid from the incoming plate were calculated to be 0.7 – 8.8 ×10^8^ kg yr^-1^ and 5.9 – 6.7 ×10^9^ kg yr^-1^, respectively.

Modeling construction

The model is formulated by a partial differential equation for solute transport and reactions^20^:

$\varphi\frac{{\partial C}_{i}}{\partial t}=D_{i}\frac{\partial}{\partial x}\left( \frac{\varphi}{\theta^{2}}\cdot\frac{{\partial C}_{i}}{\partial x} \right)-\frac{\partial{\varphi uC}_{i}}{\partial x}+{\varphi\cdot R}_{irr}+{\varphi\cdot\Sigma R}_{i}$ (S4)

where $C_{i}$ is the concentration of dissolved species ($i$) in porewater, $\varphi$ is porosity, $u$ (m yr^-1^) is upward fluid velocity, *t* (yr) is time, $x$ (m) is depth, $D_{i}$ (m^2^ yr^-1^) is the diffusion coefficient at *in situ* temperatures^20,21^ (10.5 ^o^C), $\theta^{2}(=1-\ln\left( \varphi^{2} \right)$) is the tortuosity used to correct the diffusion coefficients in porous media^20^, $R_{irr}$ is the term for bubble irrigation^22^, and ${\Sigma R}_{i}$ defines the sum of reactions occurring in the simulated sediment column. The equations were solved numerically until the model results fit the observed profiles.

To conserve the mass transport through pore, the porosity variation along depth is adopted to determine the advection rate at each depth interval^23^:

$\varphi(x)=\varphi_{f}+(\varphi_{0}-\varphi_{f})\cdot e^{-rx}$ (S5)

upward fluid velocity $u$ is calculated as^24,25^:

$u(x)=\frac{u_{0}\cdot\varphi_{0}}{\varphi(x)}$ (S6)

where $\varphi_{0}$ and $\varphi_{f}$ are the measured porosity at the sediment-seawater interface and at the depth where porosity approaches a constant value, respectively.$r$ is the porosity attenuation coefficient, which is an empirical constant obtained from data-fitting for the depth interval that covers the modeled depths. $u_{0}$ is the upward fluid velocity at the sediment-seawater interface. It is noted that the sedimentation rate^26^ (1.5×10^-3^ cm yr^-1^) is much smaller than the upward advection term. Therefore, sediment compaction alone is not enough to explain the freshening trend observed from our porewater profiles.

Bubble irrigation was incorporated into the modeling, assuming a penetration depth of at least one meter^22,27^:

$R_{irr}=\alpha_{0}\cdot\frac{\exp\left( \frac{L_{irr}-x}{\alpha_{1}} \right)}{1+\exp\left( \frac{L_{irr}-x}{\alpha_{1}} \right)}\cdot\left( C_{0}-C_{i} \right)$ (S7)

where $C_{0}$ (mM) is the concentration of target solutes in the bottom water, $L_{irr}$ (m) is the depth of the bubble irrigation layer, $\alpha_{0}$ (yr^-1^) is the intensity of bubble irrigation, and $\alpha_{1}$ (m) is the parameter controlling how expeditiously this irrigation is weakened near the bottom of the irrigation zone. All these parameters were obtained by fitting our measured porewater profiles.

The compositions of porewater from the sediment surface and bottom of core were used as the upper and lower boundaries, respectively. Dirichlet boundary conditions (with fixed concentration) were applied, assuming that the input of deep fluid was always constant from the greatest depth of the model frame. The Neumann boundary condition (with no flux) was used in the lower boundary of sulfate. The depth and time grids (dx= 0.01 m and dt= 0.01 year for all sites) were determined by running the model with progressively smaller discretization until the results were numerically stable and accurate. The results of different discretization settings are shown in Fig. S6. The model was executed to reach a steady state (within 1000 years simulation time) with initial conditions set as seawater composition.

In cold seep environments, sulfate is primarily consumed by organic matter (organoclastic sulfate reduction, OSR) and anaerobic oxidation of methane (AOM). Methanogenesis (ME) occurs below the sulfate-methane transition zone (SMTZ). Therefore, microbial reactions involving sulfate and methane were considered^25^:

${\Sigma R}_{CH4}=K_{G}\cdot\frac{\mathfrak{I\cdot}C_{org}}{2}\cdot\frac{K_{i}SO4}{K_{i}SO4+\left[ {SO}_{4}^{2-} \right]}-R_{AOM}$ (S8)

${\Sigma R}_{SO4}=-K_{G}\cdot\frac{{\mathfrak{I\cdot}C}_{org}}{2}\cdot\frac{\left[ {SO}_{4}^{2-} \right]}{K_{half-SO4}+\left[ {SO}_{4}^{2-} \right]}-R_{AOM}$ (S9)

Where $K_{G}$ represents the kinetic constant of organic matter degradation, $\mathfrak{I}$ is a parameter that converts carbon concentrations in units of wt.% C to mM and described as^28^:

$\mathfrak{I=}\frac{ds}{12}\times{10}^{4}\times\frac{1-\varphi}{\varphi}$ (S10)

K_half-SO4_ is the half-saturation constant for sulfate^29^ (0.5 mM); K_i_SO4, the inhibition constant for the initiation of methanogenesis, is poorly constrained in natural environments and assumed to be the same as K_half-SO4_ (0.5 mM); R_AOM_ is the reaction rate of AOM; C_org_ is the total organic carbon (TOC) content and assumed to be 0.45 wt.%, considering that TOC varied between 0.3 and 0.5 wt.% (Fig. S4); $K_{G}$ is assumed to be 10^-6^ yr^-1^, which denotes the slow degradation of organic carbon in deep sea environment^25,30^. The R_AOM_ term in equations (S8) and (S9) is described as^31^:

$R_{AOM}=R_{AOM}^{MAX}\cdot\frac{\left[ {SO}_{4}^{2-} \right]}{K_{half-SO4}+\left[ {SO}_{4}^{2-} \right]}\cdot\frac{\left[ {CH}_{4} \right]}{K_{half-CH4}+\left[ {CH}_{4} \right]}$ (S11)

where K_half-SO4_ and K_half-CH4_ are the half-saturation constants for sulfate and methane and assumed to be 0.5 and 5 mM, respectively^29,32,33^, [CH_4_] and [SO_4_^2-^] are the concentrations of dissolved methane and sulfate in the porewater, and $R_{AOM}^{MAX}$ is the theoretical maximum AOM rate obtained by fitting the sulfate profile (set as 2 mM yr^-1^).

Details of the parameters used in the modeling are shown in Table S4. The parameters for best fittings are shown in Table S5. Chloride profiles from the previous study were also modeled for comparisons^34^ (MV12-1 and MV12-A). Due to the short core recovery from site MV12-1, it is difficult to constrain the upward fluid velocity with the available observations. Therefore, physical conditions between sites MV12-1 and A2-2 were assumed to be the same to estimate the reaction rates at MV12-1. The variation of chloride concentration at site F6-3 is too small to derive the advection rate. Because of degassing during core retrieval, concentrations of methane measured in the pore fluids do not represent *in situ* concentrations. The saturation concentration of methane (CH4L) was calculated on the basis of the in situ temperature and pressure at the center sites (96.4 mM; calculated from a previous study^35^). At the upper flank (MV12-A) and lower flank (F6-3) sites, our model was able to fit the observed sulfate profile by assigning CH4L as 7 and 30 mM, respectively, mostly due to the short recovery of the core (Table S5).

Modeling sensitivity test

We modeled profiles for site A2-2 (at western center) as an example of sensitivity tests. The details of tests are described below:

1. Discretization of depth and time (dx and dt):

The depth and time (dx= 0.01 m and dt= 0.01 year for all sites) grids were determined by running the model with progressively smaller discretization until the results were numerically stable and accurate. Our results showed that the modeled profiles display almost the same trend regardless of whether dx was equal to 0.01 or 0.005 (Fig. S6a). When dt was decreased from 0.01 to 0.001, profiles of methane concentrations slightly changed, resulting in only an increase of 15 % in AOM rates (R_AOM_; Fig. S6b).

1. Considerations of reaction terms (rates of AOM and organic matter degradation) and bubble irrigation:

To better illustrate the significance of each terms, we tested our model in three cases: (a) with bubble irrigation, organic matter degradation (including OSR and ME), and AOM; (b) without bubble irrigation and organic matter degradation; (c) without bubble irrigation. Our results showed that profiles were not fitted in case (b) (Fig. S7b) or in case (c) (Fig. S7c), suggesting that bubble irrigation was vital to explain no significant variation of chloride concentration at the top intervals (0 to 280 cmbsf) (Fig. S7a).

1. Upward fluid velocity (u_0_), depth of bubble irrigation (L_irr_), two irrigation coefficients (α_1_ and α_0_), and lower boundary of methane (CH4L):

Our model-derived advection rates (u_0_) were most sensitive to the changes in irrigation depth (L_irr_) with 2‒2.5 folds differences in u_0_ for only a 10% variation in L_irr_ (Fig. S8a). The variations (±10%) of L_irr_ changed the AOM rate by 2 to 3% (Fig. S8b). If L_irr_ was half of the best fitting value (the best value of L_irr_ is 2.8 m), the rate of AOM was shifted by 3%. When L_irr_ was increased by 50%, the AOM rate became almost twice the best fitting one; however, such condition would not occur because the theoretical limitation of L_irr_ is up to 3 m^27^. The change (±50%) of α_1_ and α_0_ resulted in 5‒6% and 2‒4% variations in rates of AOM, respectively (Figs. S8c and S8d). Modelled values for benthic methane fluxes were insensitive to variations (±50%) in u_0_ and CH4L probably because of impact of bubble irrigation (Fig. S8e). In addition, methane benthic fluxes did not vary significantly with L_irr_ (±50%).

Estimation of brackish water expelled from 13 SMVs in Taiwan

We first fitted the calculated velocities versus distance from the edge of crater ((x‒L’) in Fig. S9b) with exponential equations (Fig. S9a). Based on different (x‒L’), velocity distribution could be further integrated with different ring areas (Fig. S9b, dashed-line) using Eq. 9. Since each SMV has different diameters of its crater and cone structure, the exponential relationships derived from (x‒L’) were replaced with “Ratio” ((x‒L’)/ (L‒L’) in Fig. S8b) and shown as follow:

${velocity}_{max}=5 \left( cm {yr}^{-1} \right)\times EXP(-13\times Ratio)$ (S12)

${velocity}_{min}=2 \left( cm {yr}^{-1} \right)\times EXP(-10\times Ratio)$ (S13)

The corresponding velocity variation along the transect at each SMV was calculated. Diameters of each crater and SMV are cited from previous observations^36^. Fluxes of brackish water discharged from each SMV are shown in Table S6.

# Table S1: Activity quotient (Q) and K_sp_ for carbonate precipitation reactions at site A2-2.

|  |  | Q | | |  | K_sp_ (at 25^o^C) | | |  | Oversaturation | | |
| --- | --- | --- | --- | --- | --- | --- | --- | --- | --- | --- | --- | --- |
| Depth (m) | Temp (^o^C) | Dolomite | Mg-calcite | Calcite |  | Dolomite | Mg-calcite | Calcite |  | Dolomite | Mg-calcite | Calcite |
| 2.86 | 16.5 | 4.03E+07 | 2.47E+04 | 1.64E+03 |  | 2.85E-04 | 6.80E-06 | 9.52E-03 |  | + | + | + |
| 3.16 | 17.7 | 1.51E+07 | 2.25E+04 | 6.74E+02 |  | 2.85E-04 | 6.80E-06 | 9.52E-03 |  | + | + | + |
| 3.46 | 19.1 | 1.33E+07 | 1.39E+04 | 9.62E+02 |  | 2.85E-04 | 6.80E-06 | 9.52E-03 |  | + | + | + |
| 3.76 | 20.5 | 9.19E+06 | 8.24E+03 | 1.12E+03 |  | 2.85E-04 | 6.80E-06 | 9.52E-03 |  | + | + | + |
| 4.06 | 22.1 | 8.08E+06 | 7.44E+03 | 1.09E+03 |  | 2.85E-04 | 6.80E-06 | 9.52E-03 |  | + | + | + |
| 4.36 | 23.8 | 4.02E+06 | 5.53E+03 | 7.26E+02 |  | 2.85E-04 | 6.80E-06 | 9.52E-03 |  | + | + | + |

Note: + represents oversaturation with respect to specific carbonate.

# Table S2: Porosity extrapolation derived from the IODP 358.

|  |  | porosity at depths (m) | | | | |  |
| --- | --- | --- | --- | --- | --- | --- | --- |
| site | $r$ | 0 m | 500 m | 1000 m | 3200 m | 5700 m | reference |
| IODP 358, site C0002^a^ | 0.00043 | 0.650 | 0.524 | 0.423 | 0.164 | 0.056 | ^38^ |

Note: calculation follows: a: $\varphi(x)=\varphi_{0}\cdot e^{-rx}$

# Table S3: Parameters comparisons for the Nankai and Taiwan subduction systems.

| parameter | Nankai Trough^a^ | Taiwan | reference |
| --- | --- | --- | --- |
| Taper angles α (slope) and  β (subduction angle) | β = 3.4–10.8^o^ | α = 3^o^; β = 6^o^ | ^6^ |
| Surface heat flow near the trench | 90–140 mW/m^2^ | 40–130 mW/m^2^ | ^39^ |
| Thermal conductivity of incoming sediments | 1.0–1.7 W/mK | 1.2 W/mK | ^40, 41^ |
| Convergence Rate | 4.0 cm/yr | 6.6–7.6 cm/yr | ^6^ |
| Bulk density of incoming sediment at depth | 1.5–2.1 g/cm^3^ | 1.4–2.2 g/cm^3^ | ^16^ |
| Porosity | 0.65 | 0.5 | ^16^ |
| Smectite abundance in incoming sediments with z | 45% | 4.5–24.8% | ^16^ |
| Mean thickness of the subduction channel | 1000 m | 1000 m |  |
| Length of the trench for the Kumano Basin and extension of the MVs area | 100 km | 172km | ^12,13^ |
| Smectite temperature stability fields | 60–150 | 60–150 |  |
| Smectite initial water content | 20% | 10–20% | ^18^ |
| Distance from deformation front  to SMV region | 55 km | 80 km | ^42^ |
| Length of SMV region | 35 km | 41 km | ^42^ |

Note: a: parameters from Nankai Trough are cited from Menapace et al. (2017)^19^.

# Table S4. Parameters used in the numerical model.

|  |  |  | Western center | | |  | Southern center | | | |  | Upper flank |  | Lower flank |
| --- | --- | --- | --- | --- | --- | --- | --- | --- | --- | --- | --- | --- | --- | --- |
| Parameter | Symbol | Unit | A2-2 | 24^a^ | MV12-1^b^ |  | MD4-P1 | MD4-P3 | 24-2 | |  | MV12-A |  | F6-3 |
| Temperature |  | ^o^C | 10.5 |  |  |  |  |  |  | |  |  |  |  |
| Diffusion coefficient of chloride | DiCl | m^2^ yr^-1^ | 0.0448 |  |  |  |  |  |  | |  |  |  |  |
| Diffusion coefficient of sodium | DiNa | m^2^ yr^-1^ | 0.0290 |  |  |  |  |  |  | |  |  |  |  |
| Diffusion coefficient of potassium | DiK | m^2^ yr^-1^ | 0.0437 |  |  |  |  |  |  | |  |  |  |  |
| Diffusion coefficient of sulfate | DiSO4 | m^2^ yr^-1^ | 0.0231 |  |  |  |  |  |  | |  |  |  |  |
| Diffusion coefficient of methane | DiCH4 | m^2^ yr^-1^ | 0.0371 |  |  |  |  |  |  | |  |  |  |  |
| Length of model column |  | m | 4.4 | 4.4 | 4.4 |  | 3.7 | 7.15 | 3.8 | |  | 1.1 |  | 2.6 |
| Porosity at sediment surface |  | φ_0_ | 0.50 | 0.50 | 0.60 |  | 0.62 | 0.62 | 0.50 | |  | 0.54 |  | 0.42 |
| Porosity at end of column |  | φ_f_ | 0.41 | 0.41 | 0.38 |  | 0.40 | 0.42 | 0.40 | |  | 0.38 |  | 0.38 |
| Empirical coefficient for porosity fitting | γ | m^-1^ | 2 | 2 | 2 |  | 2 | 2 | 2 | |  | 3 |  | 1 |
| Cl concentration at upper boundary | ClU | mM | 530 | 550 | 535 |  | 550 | 552 | 526 | |  | 537 |  | 540 |
| Cl concentration at lower boundary | ClL | mM | 200 | 310 | 200 |  | 350 | 120 | 290 | |  | 480 |  | 530 |
| Na concentration at upper boundary | NaU | mM | 465 | 480 | 467 |  | 466 | 476 | 450 | |  | 470 |  | 465 |
| Na concentration at lower boundary | NaL | mM | 230 | 310 | 230 |  | 330 | 162 | 292 | |  | 420 |  | 455 |
| K concentration at upper boundary | KU | mM | 11 | 12 | 11 |  | 12 | 12 | 10.8 | |  | 12 |  | 11 |
| K concentration at lower boundary | KL | mM | 2.5 | 7.4 | 2.5 |  | 7.3 | 2.4 | 5.5 | |  | 10.7 |  | 10 |
| Sulfate concentration at upper boundary | SO4U | mM | 27 | 27 | 27.3 |  | 27.1 | 27.5 | | 25.7 |  | 27.5 |  | 26 |

^a^: Porosities are assumed to be the same as those for A2-2.

^b^: Parameters (core length and lower boundary conditions) are assumed to be the same as those for A2-2.

# Table S5. Best fitting of all parameters applied to reactive transport modeling.

|  |  |  | Western center | | |  | Southern center | | |  | Upper flank |  | Lower flank |
| --- | --- | --- | --- | --- | --- | --- | --- | --- | --- | --- | --- | --- | --- |
| Parameter | Symbol | Unit | A2-2 | 24 | MV12-1^*^ |  | MD4-P1 | MD4-P3 | 24-2 |  | MV12-A |  | F6-3 |
| Velocity of upward fluid | u_0_ | m yr^-1^ | 0.02 | 0.03 | 0.02 |  | 0.02 | 0.05 | 0.02 |  | 0.004 |  | 0.00 |
| Depth of bubble irrigation | L_irr_ | m | 2.80 | 1.20 | 1.30 |  | 2.70 | 2.65 | 1.50 |  | 0.80 |  | 2.30 |
| Irrigation coefficient | α_0_ | yr^-1^ | 0.55 | 0.30 | 0.20 |  | 0.20 | 0.30 | 0.25 |  | 0.80 |  | 0.20 |
| Irrigation coefficient | α_1_ | m | 0.10 | 0.65 | 0.10 |  | 0.05 | 0.28 | 0.15 |  | 0.05 |  | 0.05 |
| Maximum AOM rate | $R_{AOM}^{MAX}$ | mM yr^-1^ | 2 | 2 | 2 |  | 2 | 2 | 2 |  | 2 |  | 2 |
| Methane concentration at lower boundary | CH4L | mM | 96.4 | 96.4 | 96.4 |  | 96.4 | 96.4 | 96.4 |  | 7 |  | 30 |

^*^: Parameters (core length and lower boundary conditions) are assumed to be the same as those for site A2-2.

# Table S6: Areas of craters and from flank to periphery, and fluid discharge fluxes for individual SMVs off southwestern Taiwan.

| SMV name | Crater | | |  | Flank to periphery | | |
| --- | --- | --- | --- | --- | --- | --- | --- |
|  | Area (m^2^) | Water discharge (kg yr^-1^) | |  | Area (m^2^) | Water discharge (kg yr^-1^) | |
|  |  | Min | Max |  |  | Min | Max |
| MV1 | 3.53E+03 | 2.54E+04 | 6.35E+04 |  | 1.76E+06 | 3.31E+05 | 5.64E+05 |
| MV2 | 4.84E+03 | 3.48E+04 | 8.71E+04 |  | 3.88E+05 | 1.26E+05 | 2.46E+05 |
| MV3 | 2.21E+03 | 1.59E+04 | 3.97E+04 |  | 3.14E+06 | 5.02E+05 | 8.15E+05 |
| MV4 | 6.07E+04 | 4.37E+05 | 1.09E+06 |  | 3.24E+06 | 1.27E+06 | 2.56E+06 |
| MV5 | 2.19E+04 | 1.58E+05 | 3.94E+05 |  | 3.44E+06 | 8.70E+05 | 1.60E+06 |
| MV6 | 2.90E+04 | 2.09E+05 | 5.23E+05 |  | 2.63E+06 | 8.19E+05 | 1.58E+06 |
| MV7 | 1.39E+03 | 9.98E+03 | 2.49E+04 |  | 7.84E+05 | 1.44E+05 | 2.43E+05 |
| MV8 | 6.50E+03 | 4.68E+04 | 1.17E+05 |  | 3.15E+05 | 1.30E+05 | 2.63E+05 |
| MV9 | 3.53E+03 | 2.54E+04 | 6.35E+04 |  | 8.04E+06 | 1.22E+06 | 1.96E+06 |
| MV10 | 3.53E+03 | 2.54E+04 | 6.35E+04 |  | 9.08E+06 | 1.37E+06 | 2.18E+06 |
| MV11 | 1.23E+04 | 8.84E+04 | 2.21E+05 |  | 7.06E+06 | 1.29E+06 | 2.18E+06 |
| TY1 | 1.96E+05 | 1.41E+06 | 3.53E+06 |  | 9.98E+06 | 4.00E+06 | 8.10E+06 |
| MV13 | 5.94E+04 | 4.28E+05 | 1.07E+06 |  | 3.15E+06 | 1.21E+06 | 2.44E+06 |
|  |  |  |  |  |  |  |  |
| Total |  | 2.92E+06 | 7.29E+06 |  |  | 1.04E+07 | 1.74E+07 |


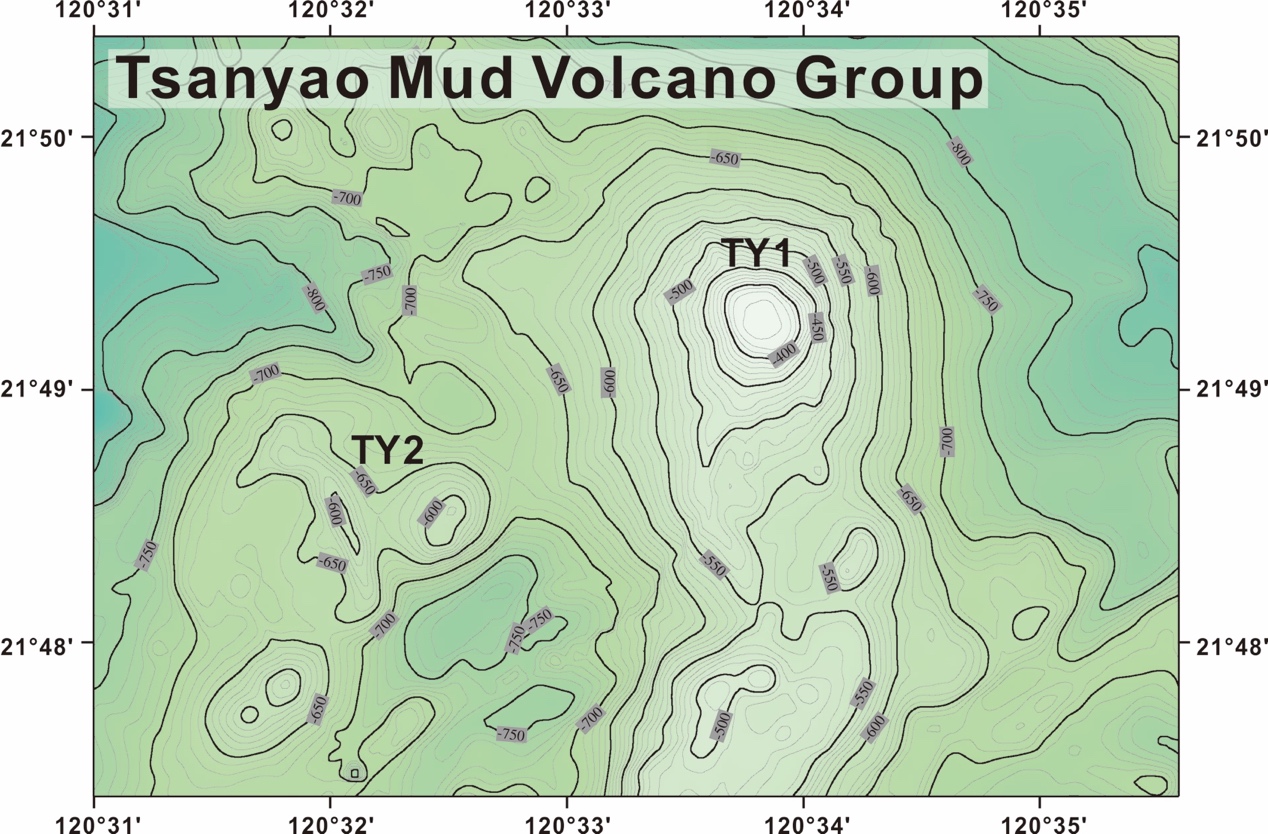


# Figure S1. Bathymetric map of Tsanyao Mud Volcano Group.


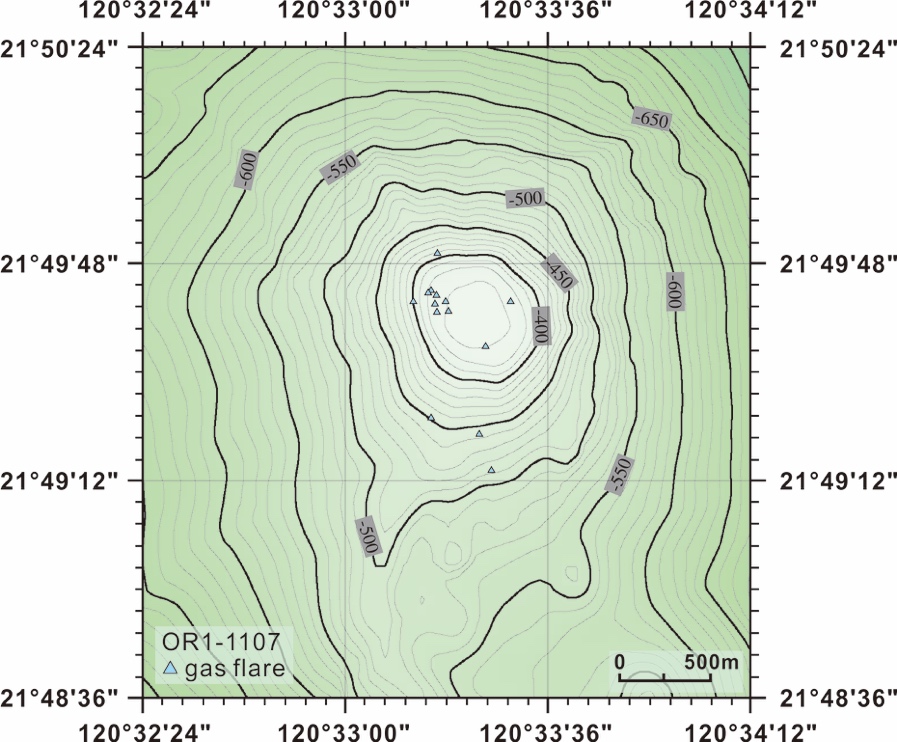


# Figure S2. Distribution of gas flares (triangles) found on TY1 during the cruise OR1-1107.


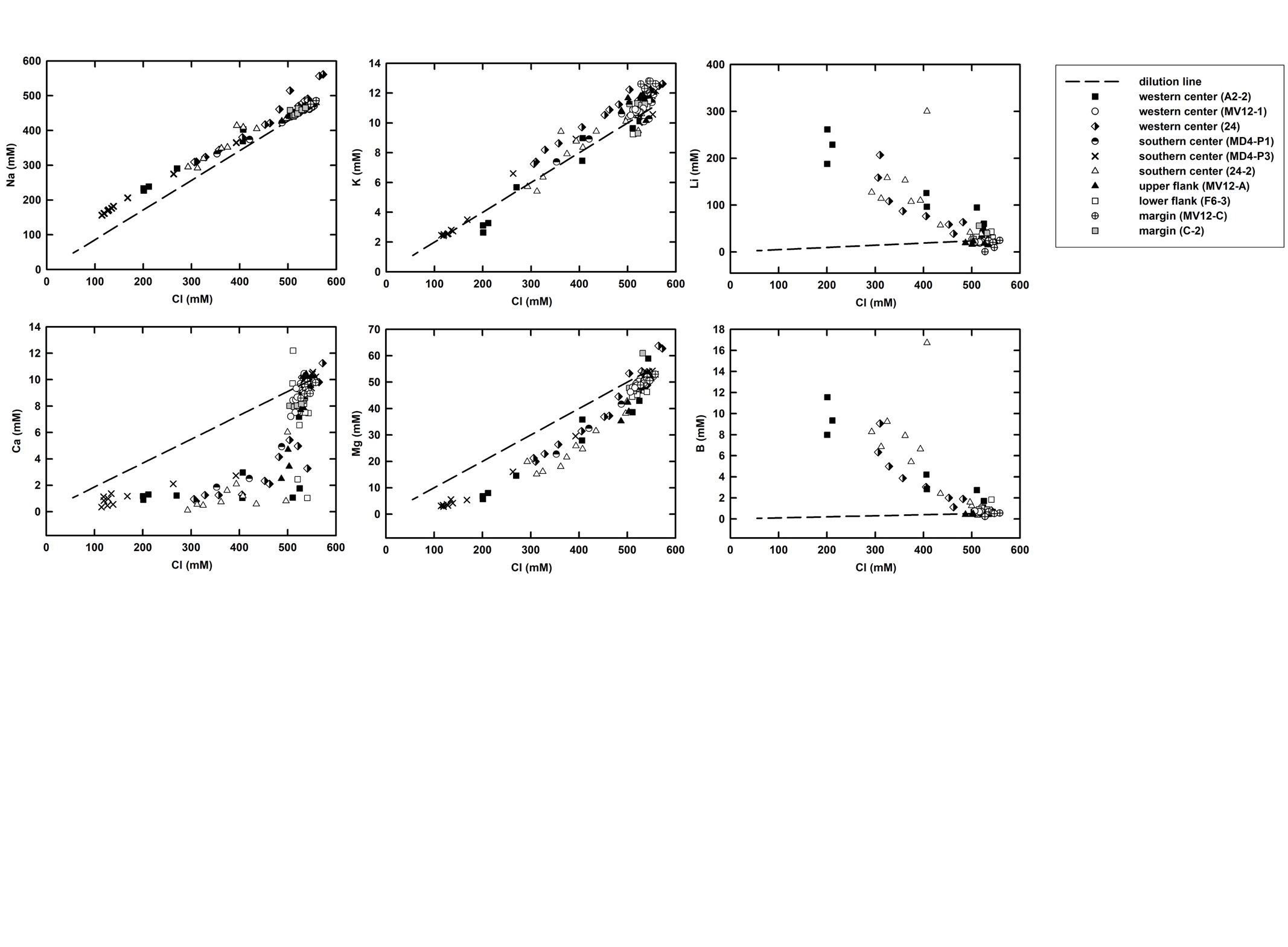


# Figure S3. Concentrations of sodium, potassium, calcium, magnesium, lithium, and boron versus chloride. The dashed-line denotes the equal dilution trend between specific ion and chloride.


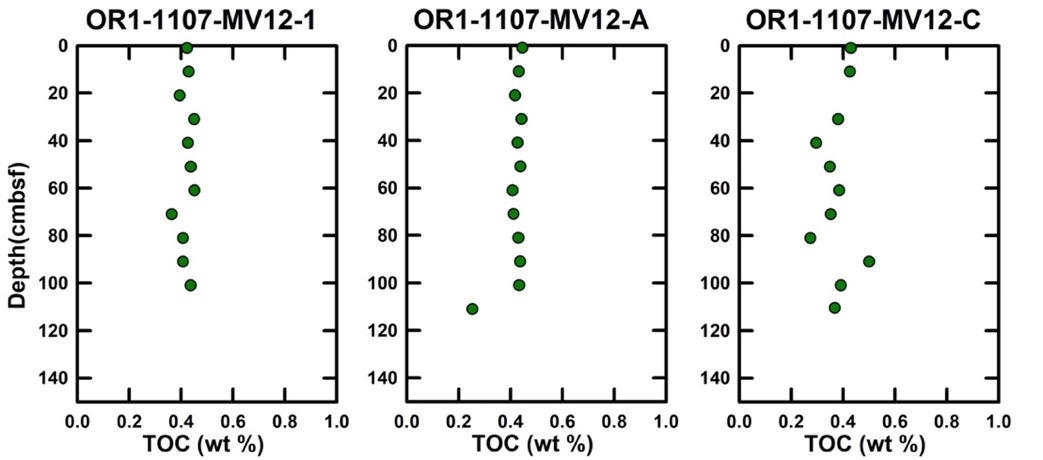


# Figure S4. TOC concentration (wt%) depth profiles at TY1.


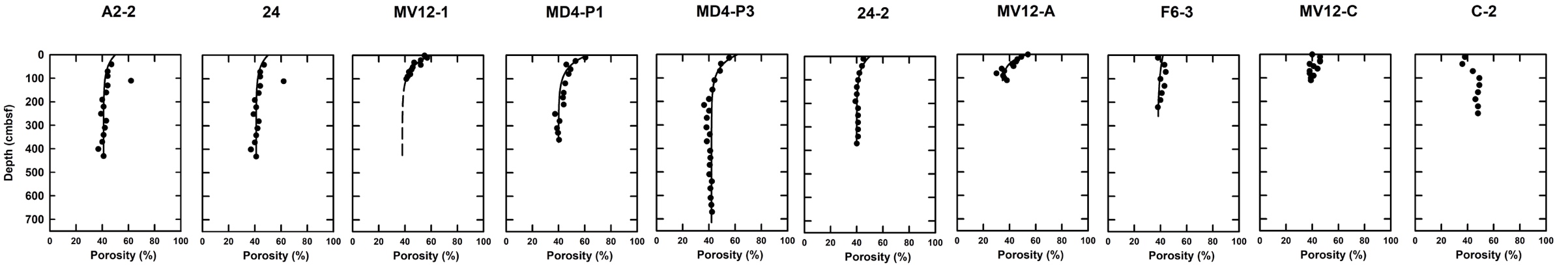


# Figure S5. Observed and modeled (black line) porosity variation with depth. Without real data, the porosity of site 24 was assumed to be the same as that of site A2-2.


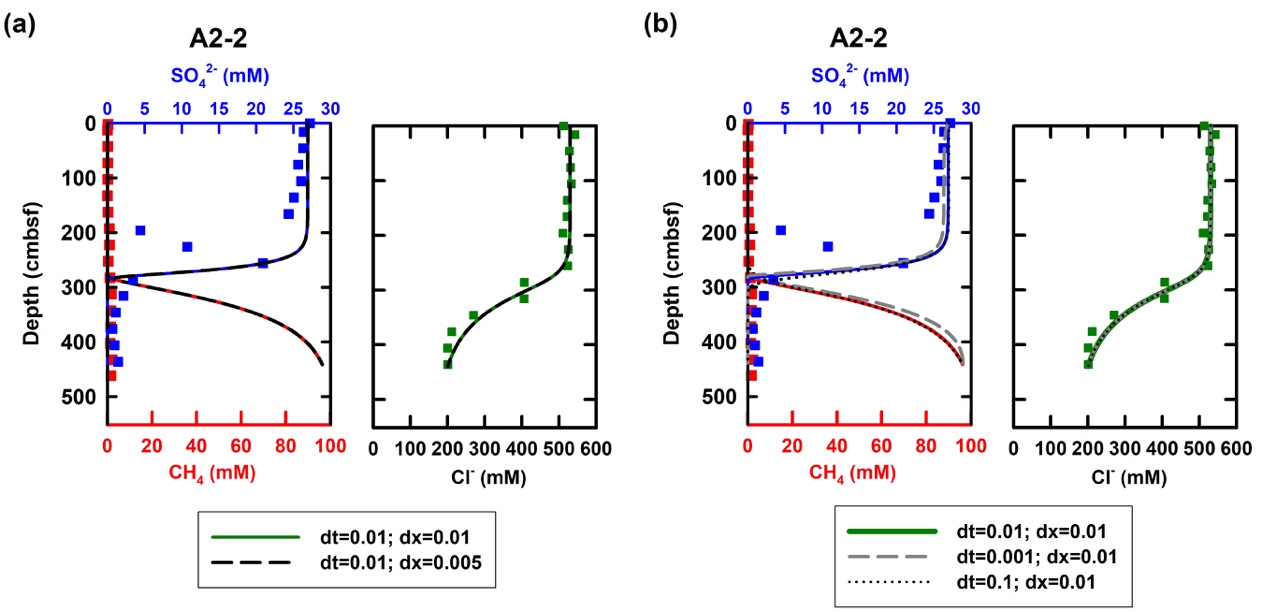


# Figure S6. Results of reactive-transport model based on different combinations of time and depth discretization.


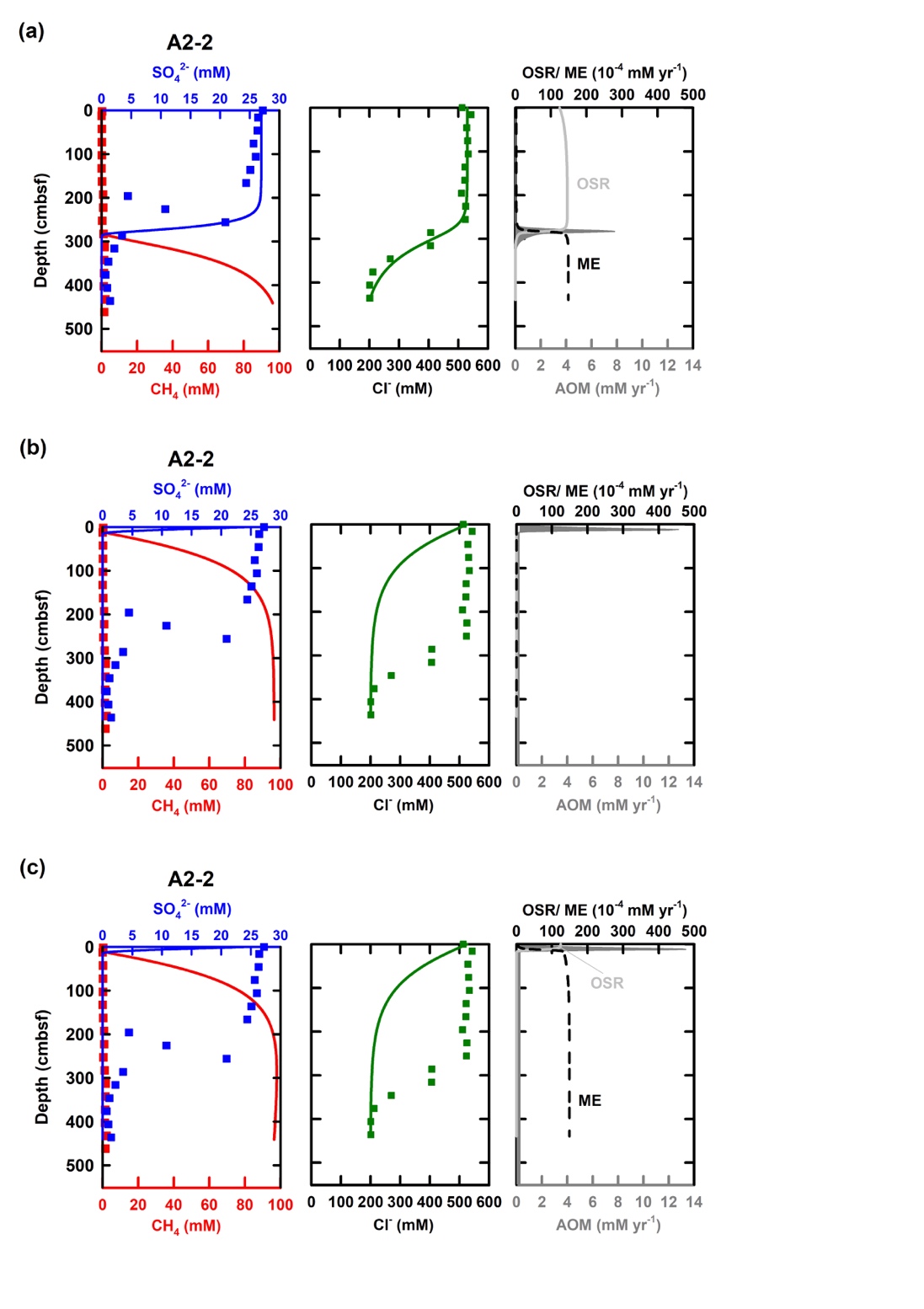


# Figure S7. Three scenarios for model test: (a) with bubble irrigation, organic matter degradation (OSR+ME), and AOM; (b) without bubble irrigation and organic matter degradation; (c) without bubble irrigation.


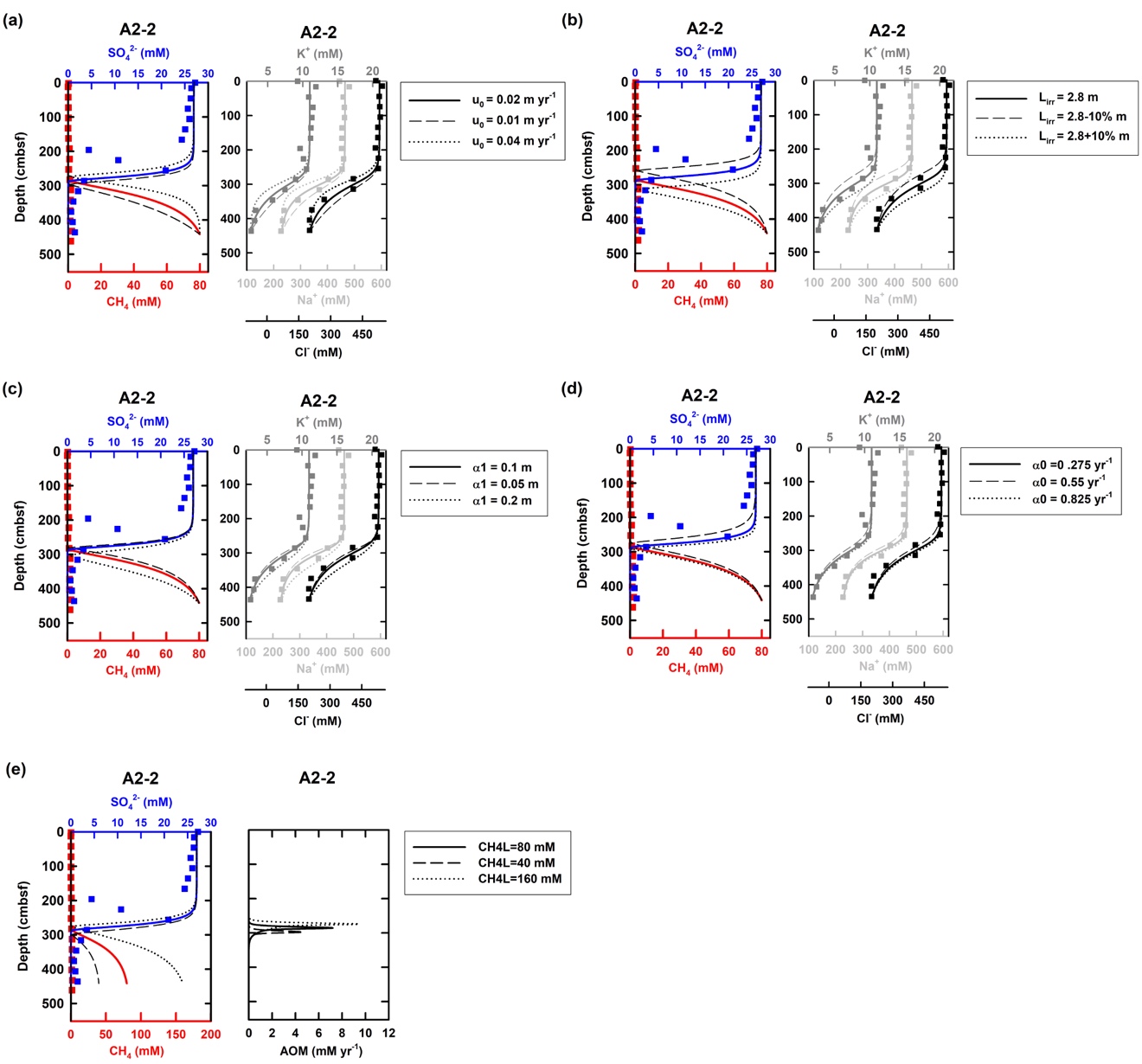


# Figure S8. Results for sensitivity tests of: (a) upward fluid velocity (u_0_); (b) depth of bubble irrigation (L_irr_); (c) two irrigation coefficients (α_1_ and α_0_); (d) lower boundary of methane (CH4L). The best fit between modeling and analyzed data is denoted by solid line.


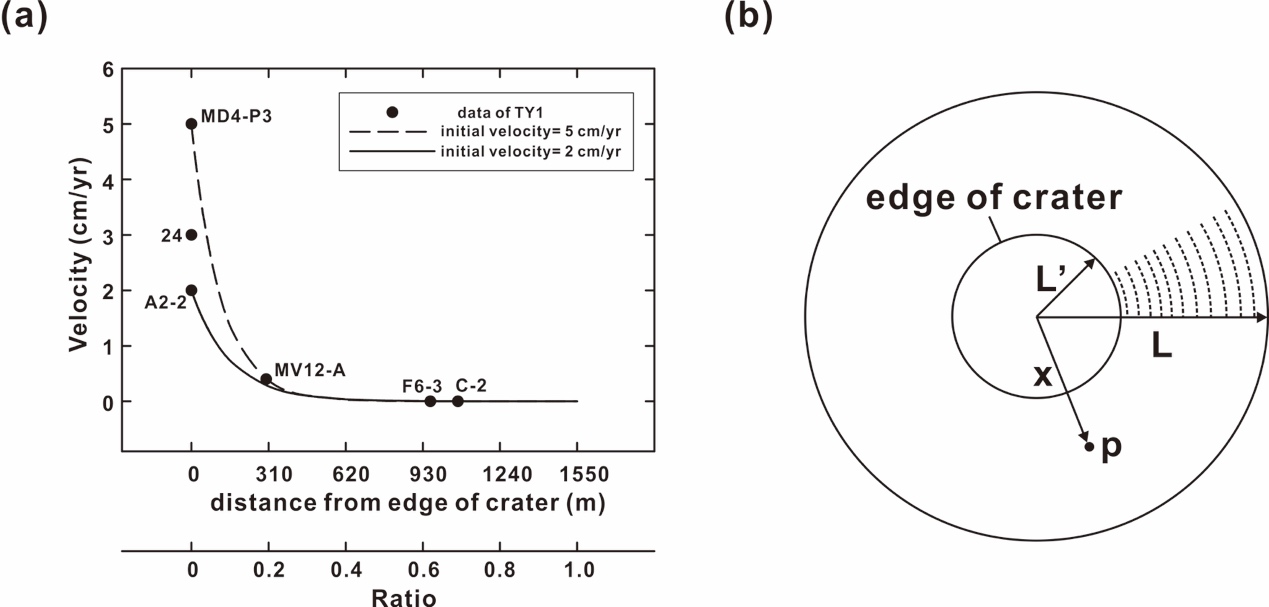


# Figure S9. (a) Exponential relationship between velocity and distance from edge of crater (x in Fig. S8b) at TY1. (b) A conceptual sketch of plane view of TY1. L is the radius of the mud volcano; L’ is the radius of crater; x is the distance between center and point p, which denotes any location between edge of crater and periphery. “Ratio” in Fig. S9a is derived from (x‒L’)/ (L‒L’).

References

1. Manov, G. G. *et al.* Values of the constants in the Debye—Hückel equation for activity coefficients. *J. Am. Chem. Soc.*, **65**(9), 1765–1767 (1943).
2. Wu, J. X. New data processing algorithm for marine heat flow and thermal modeling for Tsan-Yao mud volcano offshore SW Taiwan. Master thesis, Institute of Oceanography College of Science, National Taiwan University (in Chinese with English abstract); 10.6342/NTU201602190 (2016).
3. Lundberg, N., Reed, D. L., Liu, C. S., & Lieske Jr, J. Forearc-basin closure and arc accretion in the submarine suture zone south of Taiwan. *Tectonophysics*, **274**(1–3), 5–23; 10.1016/S0040-1951(96)00295-8 (1997).
4. Miyazaki, S. I. & Heki, K. Crustal velocity field of southwest Japan- Subduction and arc-arc collision, *J. Geophys. Res. Solid Earth*, **106**(B3), 4305–4326; 10.1029/2000JB900312 (2001).
5. Seno, T., Stein, S., & Gripp, A. E. A model for the motion of the Philippine Sea plate consistent with NUVEL- and geological data. *J. Geophys. Res. Solid Earth*, **98**(B10), 17941–17948; 10.1029/93JB00782 (1993).
6. Suppe, J. Mechanics of mountain building and metamorphism in Taiwan. *Memoir of the Geological Society of China*, **4**(6), 67–89 (1981).
7. Freundt, A. *et al.* Volatile (H_2_O, CO_2_, Cl, S) budget of the Central American subduction zone. *International Journal of Earth Sciences*, **103**(7), 2101–2127; 10.1007/s00531-014-1001-1 (2014).
8. Jarrard, R. D. Subduction fluxes of water, carbon dioxide, chlorine, and potassium. *Geochem. Geophys. Geosyst.*, **4**(5); 10.1029/2002GC000392 (2003).
9. von Huene, R. & D. W. Scholl Observations at convergent margins concerning sediment subduction, subduction erosion, and the growth of continental crust. *Rev. Geophys.*, **29**(3), 279–316; 10.1029/91RG00969 (1991).
10. Chen, M. P. Geotechnical properties of sediments off the coast of Hsinchu-northwest Taiwan related to sedimentary environment. *Acta Oceanogr. Taiwan.*, 12, 28–53 (1981).
11. Wang, P. *et al.* Exploring the Asian monsoon through drilling in the South China Sea. In *Proceedings of the Ocean Drilling Program: Initial Report* (Vol. 184, pp. 1-77); 10.2973/odp.proc.ir.184.2000 (2000).
12. Chi, W. C. *et al.* Tectonic wedging along the rear of the offshore Taiwan accretionary prism. *Tectonophysics*, **374**(3), 199–217; 10.1016/j.tecto.2003.08.004 (2003).
13. Huang, C. Y., Yuan, P. B., & Tsao, S. J. Temporal and spatial records of active arc continent collision in Taiwan: A synthesis. *Geol. Soc. Am. Bull.*, **118**, 274–288; 10.1130/B25527.1 (2006).
14. Liu, Z. *et al.* Clay mineral assemblages in the northern South China Sea: implications for East Asian monsoon evolution over the past 2 million years. *Mar. Geol.*, **201**(1), 133–146; 10.1016/S0025-3227(03)00213-5 (2003).
15. Wan, S., Li, A., Clift, P. D., & Stuut, J. B. W. Development of the East Asian monsoon: mineralogical and sedimentologic records in the northern South China Sea since 20 Ma. *Palaeogeogr., Palaeoclimatol., Palaeoecol.*, **254**(3–4), 561–582; 10.1016/j.palaeo.2007.07.009 (2007).
16. Wang, P. *et al.* Exploring the Asian monsoon through drilling in the South China Sea. In *Proceedings of the Ocean Drilling Program: Initial Report* (Vol. 184, pp. 1-77); 10.2973/odp.proc.ir.184.2000 (2000).
17. Hu, D. *et al.* Deep sea records of the continental weathering and erosion response to East Asian monsoon intensification since 14 ka in the South China Sea. *Chem. Geol.*, **326**, 1–18 (2012).
18. Bird, P. Hydration-phase diagrams and friction of montmorillonite under laboratory and geologic conditions, with implications for shale compaction, slope stability, and strength of fault gouge. *Tectonophysics*, **107**(3–4), 235–260; 10.1016/0040-1951(84)90253-1 (1984).
19. Menapace, W., Völker, D., Kaul, N., Tryon, M. D., & Kopf, A. J. The role of mud volcanism and deep‐seated dewatering processes in the Nankai Trough accretionary prism and Kumano Basin, Japan. *Geochem. Geophys. Geosyst.*, **18**(7), 2486–2509; 10.1002/2016GC006763 (2017).
20. Boudreau, B. P. Diagenetic models and their implementation, vol. 505, 132 pp. Springer, Berlin (1997).
21. Wallmann, K. et al. Kinetics of organic matter degradation, microbial methane generation, and gas hydrate formation in anoxic marine sediments. *Geochim. Cosmochim. Acta*, **70**(15), 3905–3927; 10.1016/j.gca.2006.06.003 (2006).
22. Chuang, P. C. *et al.* Relating sulfate and methane dynamics to geology: the accretionary prism offshore SW Taiwan. *Geochem. Geophys. Geosyst.*, **14**(7), 2523–2545; 10.1002/ggge.20168 (2013).
23. Boudreau, B. P. The diffusive tortuosity of fine-grained unlithified sediments. *Geochim. Cosmochim. Acta*, **60**(16), 3139–3142; 10.1016/0016-7037(96)00158-5 (1996).
24. Berner, R. A. *Early Diagenesis—A Theoretical Approach*, Princeton University Press, Princeton, N. J. (1980).
25. Vanneste, H. *et al.* Spatial variation in fluid flow and geochemical fluxes across the sediment–seawater interface at the Carlos Ribeiro mud volcano (Gulf of Cadiz). Geochim. Cosmochim. Acta, **75**(4), 1124–1144; 10.1016/j.gca.2010.11.017 (2011).
26. Su, C. C., Hsu, S. T., Hsu, H. H., Lin, J. Y., & Dong, J. J. Sedimentological characteristics and seafloor failure offshore SW Taiwan. *Terr. Atmos. Ocean. Sci.*, **29**(1); 10.3319/TAO.2017.06.21.01 (2018).
27. Haeckel, M., Boudreau, B. P., & Wallmann, K. Bubble-induced porewater mixing: A 3-D model for deep porewater irrigation. *Geochim. Cosmochim. Acta*, **71**(21), 5135–5154, doi: 10.1016/j.gca.2007.08.011 (2007).
28. Burdige, D. J., Komada, T., Magen, C., & Chanton, J. P. Carbon cycling in Santa Barbara Basin sediments: A modeling study. *J. Mar. Res.*, **74**(3), 133–159; 10.1357/002224016819594818 (2016).
29. Wegener, G. & Boetius, A. An experimental study on short-term changes in the anaerobic oxidation of methane in response to varying methane and sulfate fluxes. *Biogeosciences*, **6**, 867–876; 10.5194/bg-6-867-2009 (2009).
30. Middelburg, J. J. A simple rate model for organic matter decomposition in marine sediments. *Geochim. Cosmochim. Acta*, **53**(7), 1577–1581; 10.1016/0016-7037(89)90239-1 (1989).
31. Hong, W. L. *et al.* Seepage from an arctic shallow marine gas hydrate reservoir is insensitive to momentary ocean warming. *Nat. commun.*, **8**, 15745; 10.1038/ncomms15745 (2017).
32. Nauhaus, K., Boetius, A., Krüger, M., & Widdel, F. In vitro demonstration of anaerobic oxidation of methane coupled to sulphate reduction in sediment from a marine gas hydrate area. *Environ. Microbial.*, **4**(5), 296–305, doi:10.1046/j.1462-2920.2002.00299.x (2002).
33. Vavilin, V. A. Estimating changes of isotopic fractionation based on chemical kinetics and microbial dynamics during anaerobic methane oxidation: apparent zero-and first-order kinetics at high and low initial methane concentrations. *Antonie van Leeuwenhoek*, **103**(2), 375–383; 10.1007/s10482-012-9818 (2013).
34. Chen, N. C. *et al.* Production, consumption, and migration of methane in accretionary prism of southwestern Taiwan. *Geochem. Geophys. Geosyst.*, **18**(8), 2970–2989; 10.1002/2017GC006798 (2017).
35. Tishchenko, P., Hensen, C., Wallmann, K., & Wong, C. S. Calculation of the stability and solubility of methane hydrate in seawater. *Chem. Geol.*, **219**(1–4), 37–52; 10.1016/j.chemgeo.2005.02.008 (2005).
36. Chen, S. C. *et al.* Distribution and characters of the mud diapirs and mud volcanoes off southwest Taiwan. *J. Asian Earth Sci.*, **92**, 201–214; 10.1016/j.jseaes.2013.10.009 (2014).
37. Xie, Y. *et al*. Sediment compaction and pore pressure prediction in deepwater basin of the South China Sea: Estimation from ODP and IODP drilling well data. *Journal of Ocean University of China*, **17**(1), 25–34; 10.1007/s11802-018-3449-2 (2018)
38. Tobin, H. *et al.* NanTroSEIZE Plate Boundary Deep Riser 4: Nankai Seismogenic/Slow Slip Megathrust. *International Ocean Discovery Program Expedition 358 Preliminary Report*; 10.14379/iodp.pr.358.2019 (2019).
39. Chiao, L.-Y. Investigation of heat flow in gas hydrate potential area (4/4). *Report of Central Geological Survey*, 104-11-D, 82 pp. (2015)
40. Shyu, C. T. & Hung, C. Determination of Seafloor Temperatures Using Data from High-Resolution Marine He at Probes. *Terrestrial, Atmospheric and Oceanic Sciences*, **16**(1),137–153; 10.3319/TAO.2005.16.1.137(Oc) (2005).
41. Chen *et al.* Deriving regional vertical fluid migration rates offshore southwestern Taiwan using bottom-simulating reflectors. *Mar. Geophys. Res.*, **33**(4), 379–388; 10.1007/s11001-012-9162-4 (2012).
42. Lin *et al.* Tectonic features of the incipient arc-continent collision zone of Taiwan: Implications for seismicity. *Tectonophysics*, **476** (1-2), 28–42; 10.1016/j.tecto.2008.11.004 (2009).
